# Flexible High-Aspect-Ratio COF Nanofibers: Defect-Engineered Synthesis, Superelastic Aerogels, and Uranium Extraction Applications

**DOI:** 10.1007/s40820-025-01984-x

**Published:** 2026-01-12

**Authors:** Binbin Fan, Jianyong Yu, Xueli Wang, Yang Si, Peixin Tang

**Affiliations:** 1 College of Textiles, Donghua University, Shanghai, 201620 People’s Republic of China; 2 Innovation Center for Textile Science and Technology, Donghua University, Shanghai, 201620 People’s Republic of China

**Keywords:** Defect cleavage, COF nanofibers, Flexibility, Aerogels, Uranium extraction

## Abstract

The lack of macro-continuity and mechanical strength of covalent organic frameworks (COFs) has significantly limited their practical applications. Here, we propose an “alcohol-triggered defect cleavage” strategy to precisely regulate the growth and stacking of COF grains through a moderate reversed Schiff base reaction, realizing the direct synthesis of COF nanofibers (CNFs) with high aspect ratio (*L*/*D* = 103.05) and long length (> 20 μm). An individual CNF exhibits a biomimetic scale-like architecture, achieving superior flexibility and fatigue resistance under dynamic bending via a multiscale stress dissipation mechanism. Taking advantages of these structural features, we engineer CNF aerogels (CNF-As) with programmable porous structures (e.g., honeycomb, lamellar, isotropic) via directional ice-template methodology. CNF-As demonstrate 100% COF content, high specific surface area (396.15 m^2^ g^−1^) and superelasticity (~ 0% elastic deformation after 500 compression cycles at 50% strain), outperforming most COF-based counterparts. Compared with the conventional COF aerogels, the unique structural features of CNF-A enable it to perform outstandingly in uranium extraction, with an 11.72-fold increment in adsorption capacity (920.12 mg g^−1^) and adsorption rate (89.9%), and a 2.48-fold improvement in selectivity (*U*/*V* = 2.31). This study provides a direct strategy for the development of next-generation COF materials with outstanding functionality and structural robustness.

**Supplementary Information:**

The online version contains supplementary material available at 10.1007/s40820-025-01984-x.

## Highlights

- Covalent organic framework nanofibers (CNFs) with biomimetic scale-like architecture, record-high aspect ratio (*L*/*D* = 103.05), and superior flexibility were directly synthesized via defect engineering.
- Self-standing membranes and nanofibrous aerogels (CNF-As) with designable micro-topological structures were fabricated with 100% CNFs.
- CNF-As perform photo-induced uranium extraction with an adsorption capacity and adsorption rate of 920.12 mg g^−1^ and 89.9%, respectively.
- CNF-As exhibit superior underwater stability (> 180 days) and superelasticity (~ 0% deformation after 500 compression cycles), making them promising for practical application in marine systems.

**Supplementary Information:**

The online version contains supplementary material available at 10.1007/s40820-025-01984-x.

## Graphical abstract

**Figure:**
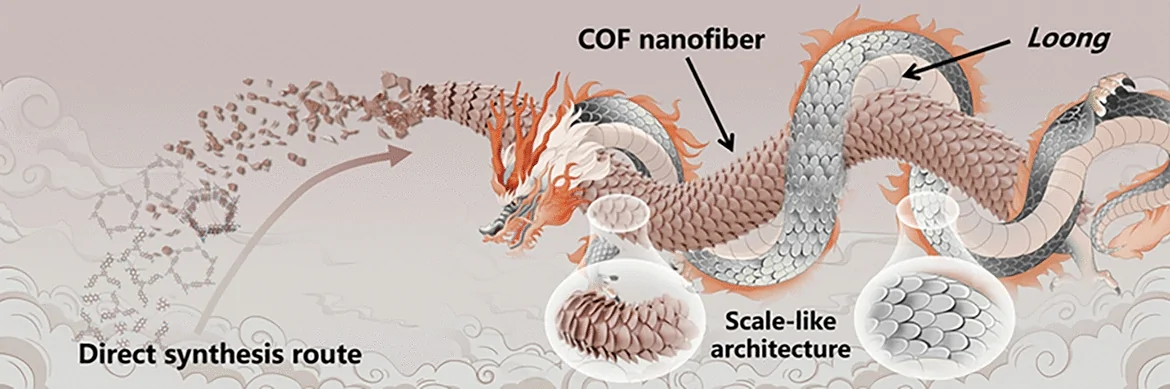

## Introduction

Covalent organic frameworks (COFs) have emerged as a novel class of crystalline porous polymers for advanced molecular technologies due to high specific surface area (SSA), abundant micro/mesopores, and designable functionalities. However, their transition from particulate powders to macroscopic functional materials remains impeded by intrinsic brittleness and structural discontinuity [1–5]. While composite engineering through substrate hybridization offers partial solutions, compromised active site accessibility and interfacial stress concentration fundamentally limit their practical performances [6–10]. Moreover, achieving stable and massive integration with the supporting substrate in the composites necessitates precise control of COF particle sizes at the nanoscale with high uniformity, which is a persistent challenge in solvothermal synthesis. Thus, attempts have been made to increase the COF dimensions and to directly shape them into macro-continuous materials such as self-standing films and monoliths. Although interfacial synthesis enables the fabrication of COF films, their structural integrity critically depends on the chemical composition of the COF and synthetic parameters, deviations from optimal conditions usually result in crack formation [11–13]. The dense packing of COF grains in the vertical direction (*Z*-axis) in the film inherently limits their application performances. Keeping this challenge in mind, COF aerogels with 3D porous architecture have been engineered through a sol–gel strategy coupled with supercritical CO_2_ drying [14–16]. Nevertheless, these aerogels possess irregular porous structures that resist precise manipulation or pre-fabrication design. More critically, most reported COF aerogels demonstrate insufficient elasticity and restricted structural adaptability, which are fundamental drawbacks impeding practical application.

Fibers, with their high aspect ratio (*L*/*D*) and capability for textile design, represent a unique material form that combines structural adaptability and multifunctional synergy. Crystalline materials exhibit exceptional mechanical robustness and physiochemical properties due to ordered lattice arrangements, but their inherent brittleness constrains their utility in flexible devices. Emerging strategies to circumvent these limitations focus on engineering crystalline materials into fibrous morphologies by controlling crystallization dynamics and microstructural topology. The one-dimensional continuity of fibers enables precise regulation of stress distribution via lattice orientation and defect engineering. A notable example includes the puzzle-like polycrystalline stacking in oxide ceramic crystal nanofibers, which balances strength and flexibility through grain boundary sliding and localized energy dissipation under strain [17]. Recent advances have extended this strategy to COFs, with scientists developing methods to shape COFs into a fibrous architecture. For instance, Ma et al. [18] designed a solvent-mediated approach to promote the growth of COF along the (001) crystal plane to form crystalline nanowires with lengths greater than 2 μm and *L*/*D* ranging from 10 to  40. Pan et al. [19] regulated the interfacial interactions between COF sheets, resulting in the disintegration of COF from lamellar structures into fibrous morphologies, whose lengths and *L*/*D* ranged from 3 ~ 5 μm and 15 ~ 25, respectively. In addition, Wang et al. [20] achieved the transformation of COF particles to nanofibers by regulating the reversible condensation-termination reaction in COF synthesis, thus achieving nanofibers with lengths of > 20 μm and L/D ranged from 30.8 to 66.7. However, the insufficient *L*/*D* value (< 100) of such COFs makes them possess weak mechanical strength and interfiber entanglement, posing difficulties in direct fabrication of macro-continuous materials. Currently, the interdisciplinary integration between COF chemistry and fiber science remains insufficient, resulting in inadequate attention to purposely regulate the COF morphology into fibers with high aspect ratio. Mechanisms governing morphological evolution of COFs into fibers remain unclear, thereby limiting the production of COFs with sufficient aspect ratio and length for macro-continuous material fabrication.

Scale architectures represent remarkable biomechanical advantages evolved to reconcile structural rigidity with dynamic flexibility, which could be commonly observed in organisms such as snakes and pangolin in nature, and *loong* in traditional Chinese mythology. In pangolins, for instance, keratinous scales are arranged in imbricated patterns across the dermal surface, with specialized micro-hinge joints connecting adjacent scales. The scale-hinge architecture dissipates external mechanical energy by coordinating microstructural deformation, *i.e.*, scale sliding and rotational freedom at hinge junctions during flexion, to achieve local stress redistribution across multilayered assemblies, thereby enabling fracture resistance and macroscopic flexibility simultaneously. While scale structures are common for bio-organisms, it has shown great challenges to be achieved in synthetic materials.

Herein, we report an “alcohol-triggered defect cleavage” strategy to synthesize COFs into nanofibers (CNFs) with a scale-like morphology, thus realizing enhanced structural stability and flexibility of COFs. 2,6-Diaminoanthraquinone (DAAQ) and 2,4,6-triformylphloroglucinol (TP) are selected as monomers for their ability to form photosensitive COFs. Benzyl alcohol (BA), acting as a moderate nucleophile, facilitates cleavage and reorganization of COF grains through reversible Schiff base reaction, ultimately leading to the formation of flexible CNFs with hierarchical structures. The resulting CNFs exhibit a record-high *L*/*D* of 103.05 with a length of > 20 μm and a wool-like elasticity (elastic modulus ~ 4.90 GPa), enabling their assembly into self-standing membranes and aerogels without other supporting polymers. As a proof-of-concept study, CNFs are constructed into nanofibrous aerogels (CNF-As) with precisely controlled porous structure (e.g., honeycomb, lamellar, and isotropic), high SSA (396.15 m^2^ g^−1^), and superior mechanical stability and elasticity (~ 0% deformation after 500 compression cycles). Given that CNF-As are constructed from 100% nanofibrous COFs, its application performances, such as photo-induced uranium extraction, are significantly better than the COF particle-embedded aerogels. The successful synthesis of CNFs not only addresses the inherent brittleness and processability issues of conventional COFs but also opens new avenues for their application in macro-continuous functional materials such as films, membranes, and aerogels.

## Experimental Section

### Materials

2,4,6-Triformylphloroglucinol (TP, 97% NMR, Energy Chemical), 2,6-diaminoanthraquinone (DAAQ, 98%, Adamas-bata), acetic acid (99.7%, Aladdin), mesitylene (M, AR, 97%, MACKLIN), benzyl alcohol (BA, CP, SCR), methanol (MA, 99.5%, reagent grade), isopropyl alcohol (IPA, AR, SCR), cinnamyl alcohol (CA, 98%, Shyuanye), N, N-dimethylacetamide (DMAc, 99.0%, Boer), and menadione sodium bisulfite (MSB, 95%, Shyuanye) were supplied by Reagent Website of Donghua University.

### Direct Synthesis of CNFs

The photoactive CNF was prepared based on conventional solvothermal synthesis. Specifically, TP (0.084 g, 0.40 mmol) and DAAQ (0.143 g, 0.60 mmol) were first dissolved in M/BA (v/v = 1/1) (25 mL) separately and mixed into a 100-mL autoclave, and acetic acid (6 M, 0.3 mL) was added as the catalyst. The reaction system was purged with N_2_ gas for 10 min followed by heating the system at 120 °C for 48 h. The resultant precipitates were washed with DMAc, H_2_O, and MA. The photoactive CNFs with a yield of 94.11% were finally obtained via 60 °C vacuum dry for 12 h. It is noteworthy that the scalable production of CNFs is only limited by the size of the equipment (e.g., autoclave and oven). For instance, the production amount of CNF could achieve as 24 g per day in our laboratory.

### Fabrication of CNF-Ms and CNF-As

(i) Fabrication of CNF-M: CNFs (0.20 g) and deionized water (19.80 g) were added into a 25 mL glass beaker with menadione sodium bisulfite (0.002 g, MSB) and bacterial cellulose nanofiber suspension (solid content = 0.8%, 0.02 g, BCN) as photo-crosslinker and dispersing agent, respectively. Subsequently, the mixed solution was homogenized under ultrasonication (25 kHz, 120 W) for 5 min in a pulse mode while the temperature was maintained at 25 ± 2 °C in an ice bath. The homogenized solution was filtered under vacuum for 10 min. Following this, the PTFE filter membrane with wet CNF-M was transferred into a freeze dryer (− 60 °C, 1.0 Pa) for 48 h. Finally, the CNF-M was obtained after a UVA (365 nm, 300 mW cm^−2^) irradiation in a crosslinking box (UVGO, China) for 10 min to form chemical crosslinking between CNFs. (ii) Fabrication of CNF-As: The same homogenized solution was prepared as above. Then, the homogenized solution was transferred into customized aerogel molds followed by freezing with liquid nitrogen into ice cubes. The lyophilization and UVA crosslinking were performed on the ice cubes to finally achieve CNF-As according to the same procedures as described above.

## Results and Discussion

### Rational Design

**Fig. 1:**
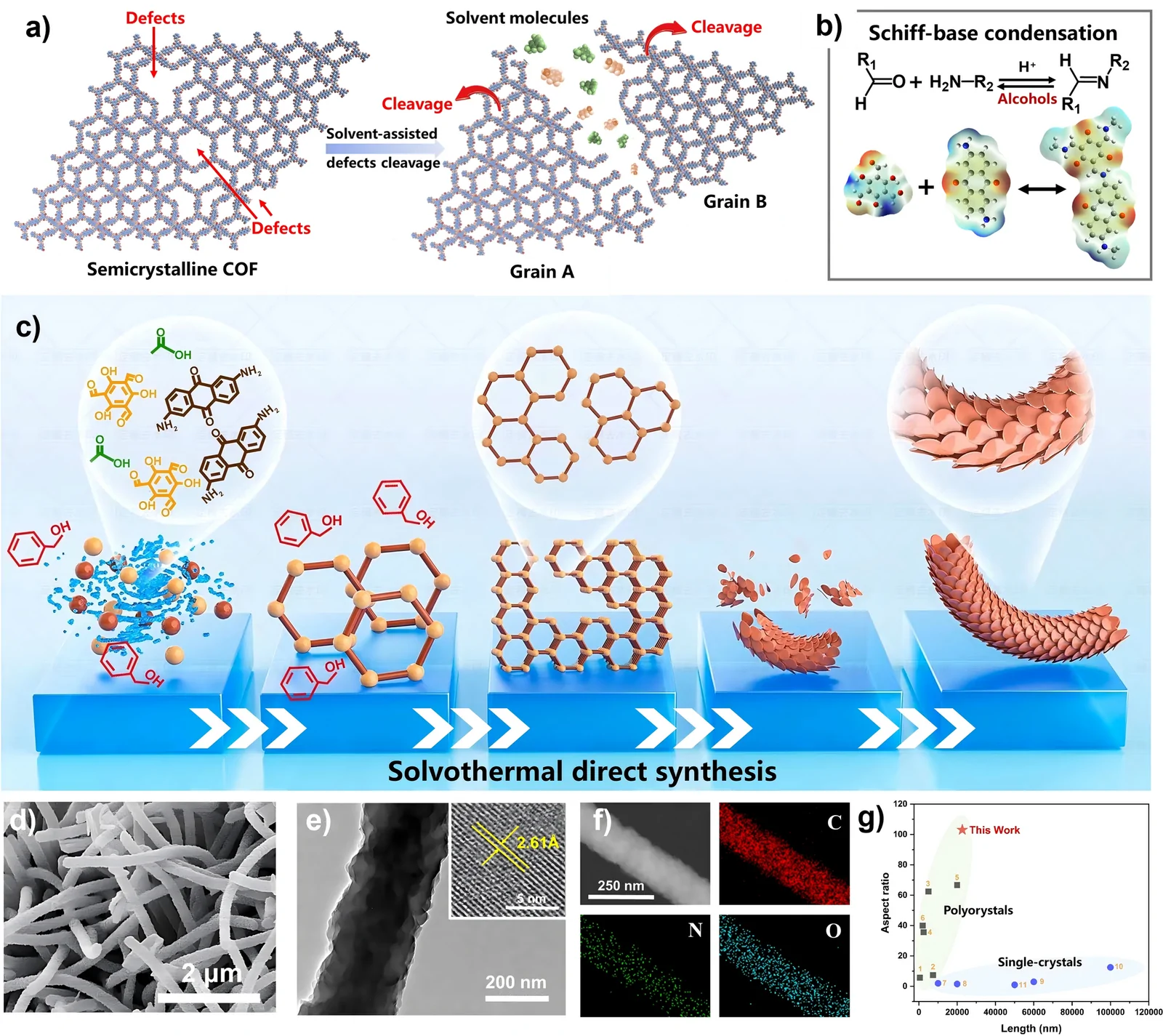
Rational design and structural demonstration. **a, b** Demonstrations of the defect cleavage process by alcohols based on a reversed Schiff base reaction. **c** Illustration of CNFs synthesis via direct solvothermal methodology. **d** SEM, **e** TEM, and **f** EDS mappings of CNFs. **g** Lengths and aspect ratios of reported anisotropic COFs obtained from solvothermal synthesis

Mimicking the scale architecture in nature, we engineer COFs into nanofibers with scale-like morphology through an “alcohol-triggered defect cleavage” strategy. We chose 2,6-diaminoanthraquinone (DAAQ) and 2,4,6-triformylphloroglucinol (TP) as monomers, and the resultant COFs are obtained via a Schiff base condensation reaction (Fig. S1). Generally, defects (*i.e.*, amorphous regions) are difficult to be avoided during the COF synthesis, and a specific reaction condition could even selectively design the COF defects, resulting in COFs with various morphologies and properties [21–25]. As proposed in Fig. 1a, b, small molecules with moderate nucleophilicity could act as defect cleavage agents to first penetrate into the defects of a semicrystalline COF, and then cleave the COF into grain A and grain B along the defects based on a reversed Schiff base reaction [20]. After the cleavage, the crystallinity of the COF increased, and its morphology could also be designed. As illustrated in Fig. 1c, benzyl alcohol (BA), a moderate nucleophile, was introduced into the synthesis system as a defect cleavage agent to manipulate the morphology of COFs into nanofibers. During the synthesis, BA will diffuse into the COF defects and break the imine bonds that are formed by the monomers. The released active groups (i.e., amine or aldehyde) from the detects could undergo the Schiff base reaction again to modify its crystalline structure. After several rounds of this bond breaking-forming process, COF grains will grow into nanoflakes, and they will finally aggregate together to produce nanofibers with a scale-like architecture. As exhibited in Fig. 1d, CNFs with an averaged diameter of 200 nm and a length over 20 μm were successfully obtained through the solvothermal synthesis without any structural supporting materials. The atomic force microscope (AFM) images also showed that the CNFs possess surface roughness, which differs from large-scale COF single crystals (Fig. S2) [26–28]. To deeply explore the microstructure of CNFs, high-resolution-transmission electron microscope (HR-TEM) was adopted. As shown in Fig. 1e, an individual CNF was constructed by multiple COF grains with misaligned overlapping, which is highly similar to the structure of biological scales. The magnified HR-TEM image of the CNF clearly showed the lattice distance of (100) as 2.61 Å. Energy-dispersive spectroscopy (EDS) was performed to examine the elemental distribution on CNFs. Figure 1f showed that carbon (C), nitrogen (N), and oxygen (O) are all uniformly distributed along the CNF, which demonstrated the successful synthesis of CNFs with high purity. The attenuated total reflection (ATR) spectra and the ^13^C solid state nuclear magnetic resonance (SSNMR) spectra provide extra structural evidence of CNF (Fig. S3).

The above results triggered us to deeply explore the formation process of CNFs, thus understanding its synthesis mechanism. By examining the morphologies of CNFs at different synthesis timepoints, we could clearly see a time-dependent morphology and crystallinity evolution (Figs. S4 and S5b). Specifically, before heating (i.e., 0 h), two monomers already formed aggregates, the sizes of which are determined by the composition of the solvent system (e.g., alcohol type, alcohol concentration, etc.) (Figs. S4a and S6). When the reaction starts, the COFs preferentially stack in a two-dimensional direction due to Schiff base condensation, gradually transforming from aggregates into uniformly shaped nanoflakes (Fig. S4b–f), then these nanoflakes continuously grew to exhibit a thickening effect when the reaction time was prolonged to 10 h (Fig. S4g). We then observed that the thickened nanoflakes started to cleave into nanofibers and presented uniform fiber diameters when the synthesis time reached 48 h (Fig. S4h–j). By further increasing the reaction time to 72 h, shorter COF nanofibers were obtained (Fig. S4k). To further elucidate the morphology evolution of CNFs in terms of defect cleavage and crystallinity variation, deep analyses of XRD spectra and the synthesis kinetics were performed. As shown in Figs. S5 and S7, the formation of CNF proceeds through four distinct stages: (1) within the first 8 h of synthesis, crystallinity increased initially due to the formation of the COFs via Schiff base condensation, consuming available monomers. (2) A sudden drop in crystallinity was observed at 10 h, coinciding with the approach of reaction equilibrium where monomer consumption and CNF yield stabilized. At this stage, the Schiff base reaction rate approaches its peak, which far exceeds the molecular ordering rate, forming disordered packing regions that subsequently trigger defect cleavage initiated by alcohols; (3) with further prolonging the reaction time, the crystallinity recovered and plateaued until 48 h, attributed to structural correction and reorientation driven by the alcohol-triggered reversed Schiff base reaction; (4) the crystallinity decreased again with increasing the reaction time to 72 h, resulting from the gradual disintegration of COF grains under prolonged heating. This hypothesis was also proved by noticing a decrease of CNF yield. Moreover, the concentration of the catalyst also poses a significant effect on the CNF morphology, which controls the kinetics of the Schiff base reaction (Fig. S8). An appropriate amount of catalyst provides sufficient time for CNF to correct and revise its structure. During the synthesis, the proportion of BA in the solvent system played a crucial role in controlling the CNF morphology (Fig. S9a–g). With the absence of BA, *i.e.*, mesitylene (M) as the solvent, particulate and low crystalline products were obtained. With increasing the volume fraction of BA, we clearly observed that the product morphology shifts to nanofibers, and the more the BA, the finer the fibers and the higher the crystallinity (Fig. S6h). Compared with the reported anisotropic COFs, including single crystals and polycrystals, CNFs that featured a record-high aspect ratio (103.05), long fiber length (> 20 μm), and uniform nanofiber architecture were achieved in our work when the synthetic conditions were solvent: mesitylene/BA = 1/1 v/v, catalyst: 6 M acetic acid, temperature: 120 °C, and time: 48 h, respectively (Fig. 1g and Table S1).

### Investigation of Synthesis Mechanism of CNFs

**Fig. 2:**
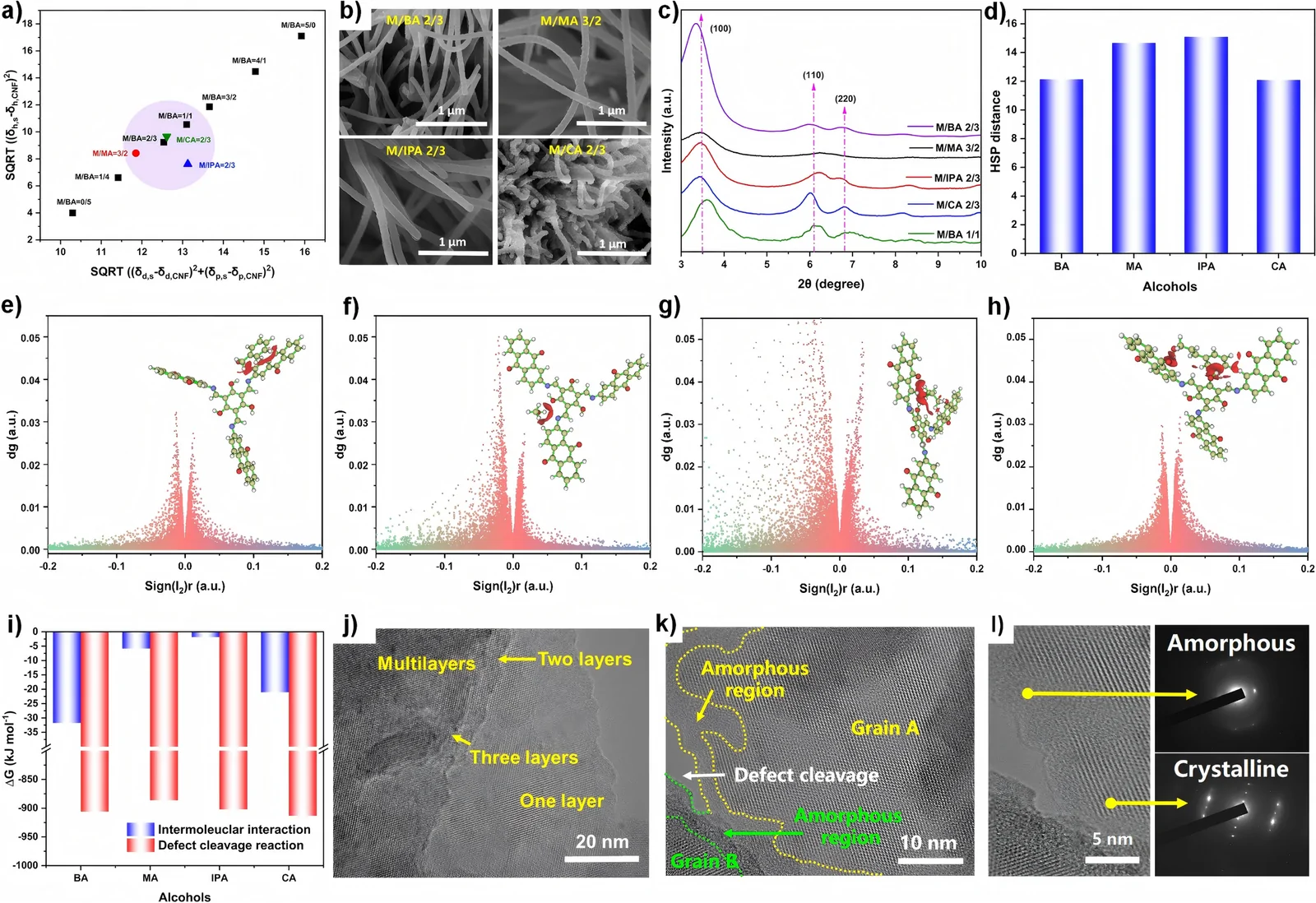
Investigation of synthesis mechanism of CNFs.** a** 2D plot with HSPs as the descriptors and locations of solvents that allows CNFs to be formed. **b** SEM images of CNFs obtained from different alcohol systems. **c** XRD spectra of CNFs synthesized from solvent systems of M/BA = 2/3 v/v, M/MA = 3/2 v/v, M/IPA = 2/3 v/v, and M/CA = 2/3 v/v. **d** HSP distance between CNF analog and different alcohols. **e–h** IGMH analysis of non-covalent interactions of CNFs with **e** BA, **f** MA, **g** IPA, and **h** CA. **i** The calculated Gibbs free energy changes (Δ*G*) of interested reactions. **j-k** HR-TEM and **l** corresponding SAED pattern of an individual CNF

To gain a deeper understanding of the role of BA, other alcohols, including methanol (MA), isopropanol (IPA), and cinnamyl alcohol (CA), were attempted for CNFs synthesis. The addition of alcohols with different polarity primarily affects the solvent properties, which influences the solubility of monomers and the resultant COFs, manipulating the morphology of as-synthesized CNFs [29]. Hansen solubility parameter (HSP) theory describes molecular interactions through contributions from three types of cohesion energies: dispersion forces (*δD*), dipole interaction (*δP*), and hydrogen bonding (*δH*) [29]. Referenced from Chen et al. [30] that the contribution to the affinity between two substances in solution from dispersion forces (*δD*) and dipole interactions (*δP*) could be integrated into one parameter. By correlating 2D HSP plots (*δD* + *δP* vs *δH*) of solvent systems to the CNF architecture (Figs. 2a, b and S6), we identify that the optimal CNF formation occurs when the HSP distances between solvent and CNF approach 15.79 ~ 16.88 MPa^1/2^ (*i.e.*, M/BA = 1/1 ~ 2/3 regimes). Intriguingly, MA/IPA/CA-involved systems only yield CNFs with good crystallinity when their HSPs converge to a critical window with |Δ*δ*| for each interaction parameter ≤ 1.83 MPa^1/2^ (Fig. 2b, c), confirming that a balanced dispersion–dipole–hydrogen interaction of the solvent system is prerequisite for CNF growth (Table S2).

The alcohol-COF interfacial affinity critically dictates defect cleavage efficiency, where the optimal HSP matching enables alcohol penetration into amorphous domains to initiate the reversed Schiff base reaction, thus determining the morphology of the CNFs. Figure 2d summarized the HSP distance between alcohols and CNF, demonstrating an order with decreased affinity as BA > CA > MA > IPA. In addition, density functional theory (DFT) and the independent gradient model based on Hirshfeld partition (IGMH) analysis were adopted to clarify their intermolecular interactions. As presented in Fig. 2e–h, BA/CNF and CA/CNF predominantly exhibited van der Waals interaction, while the MA/CNF and IPA/CNF presented strong hydrogen bond interaction (green denotes strong attraction including hydrogen bond, pink indicates van der Waals interaction, and purple-to-blue refers to strong repulsion such as steric hindrance) [31–33]. The high affinity between the alcohol and CNF was also proved by calculating the change of Gibbs free energy (Δ*G*) of forming alcohol/CNF composites. As presented in Fig. 2i, all composites show negative Δ*G*, indicating the process of composite formation is thermodynamically allowed, i.e., high affinity existed between the alcohol and CNF. According to the calculated Δ*G* values, the affinity followed an order of BA (− 31.73 kJ mol^−1^) > CA (− 21.07 kJ mol^−1^) > MA (− 5.84 kJ mol^−1^) > IPA (− 1.84 kJ mol^−1^), which is consistent with that predicted based on HSP theory (Fig. 2d).

More importantly, the cleavage of the defects is crucial for CNF to achieve its nanofibrous morphology. The “alcohol-triggered defects cleavage” via the reversed Schiff base reaction specifically involves four steps: (1) protonation of the imine-N in CNF, (2) attack of the imine bond by the alcohol, (3) proton rearrangement within the imine linkage, and (4) breakage of the imine linkage (Fig. S13–S16). As summarized in Fig. 2i, the reversed Schiff base reactions triggered by four alcohols are all spontaneous by showing an overall Δ*G* of − 837.50 ~ − 912.50 kJ mol^−1^, while thermal activation (*i.e.*, 120 °C) to overcome a 78.22 ~ 118.84 kJ mol^−1^ energy barrier at Step 3 is required (Table S3). We exploited the nucleophilicity of alcohols to shape COFs into nanofibers with a scale-like architecture via controlled grain disassembly and re-stacking, which diverges from the control of epitaxial growth mechanism that reported by Ma et al. [18].

We then carefully investigated the microstructure of CNFs using HR-TEM and the selected area electron diffraction (SAED) to provide direct evidence for the proposed mechanism. BA-engineered CNFs exhibit hierarchically ordered assemblies of COF grains through misaligned stacking without forming interstitial pores (Fig. S17). As the magnified TEM results shown in Fig. 2j, from the fiber edge to the core, COF grains with different thicknesses and number of layers can be seen clearly. Here, the BA in the synthesis system targeted to cleave the defects and acted as a grain size and crystallinity modifier, thus a large number of grains with different orientations could be observed in an individual CNF (Fig. S18). The microstructures of grain A and grain B in CNF during the cleavage are labeled in Fig. 2k, where the cleaved edges present as amorphous while their interiors show a regular lattice structure with high crystallinity (Fig. S18). The defect cleavage produces crystalline/amorphous interfaces, which are further evidenced by the SAED patterns showing crystalline cores that are surrounded by amorphous edges (width of ~ 5 nm) (Fig. 2l).

### Demonstration of the Flexibility of Individual CNFs

**Fig. 3:**
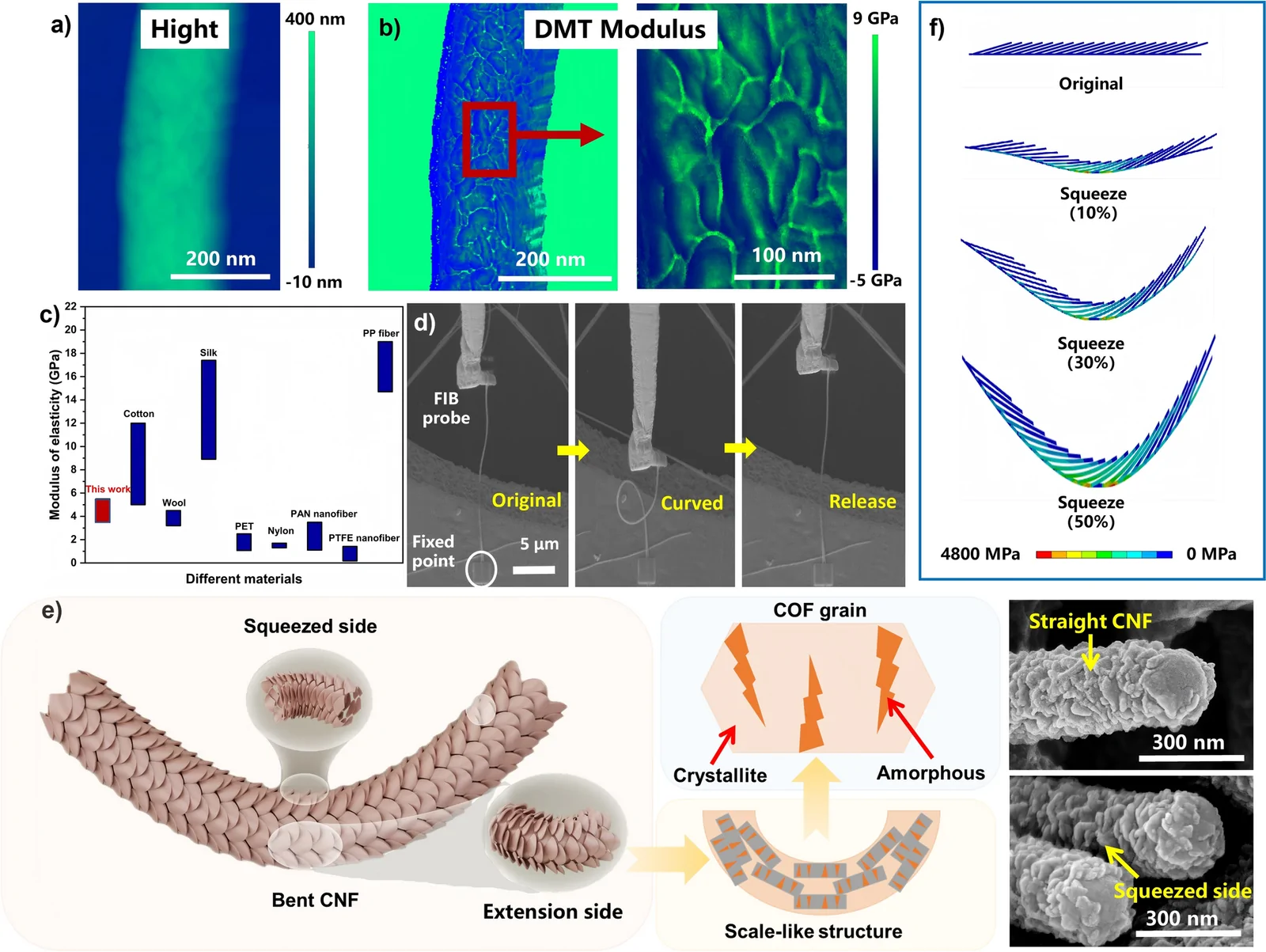
Demonstration of Flexibility of CNFs. **a** 3D AFM image and **b** elastic modulus mapping of an individual CNF. **c** Elastic modulus of CNF compared with traditional fibers. **d** Dynamic bending and recovery characterizations of an individual CNF. **e** Illustration and SEM images of a bent CNF. **f** FEA simulation of the squeezing side of the CNF under dynamic bending

The softness and flexibility of CNFs are highly related to their microstructure. The softness of nanocrystalline materials could be achieved by reducing the grain size, allowing the stress relaxation to occur at grain boundaries [34]. In addition, the gain boundaries with low crystallinity are able to scatter and dissipate the stress concentration, thus achieving bending of CNF under external stress without breakage. To explore the mechanical properties and flexibility of CNFs, AFM in peak force quantitative nanomechanics (PF-QNM) mode was adopted to monitor the individual CNF. For the sample preparation, CNFs were first ultrasonically (180 W) dispersed in ethanol for 5 min and then dropped on a mica substrate for air-dry. Then, the AFM probe scanned the CNF surface in a raster pattern, generating high-resolution 3D topographic images as shown in Figs. 3a and S2. Consistent with the SEM and TEM results, CNF exhibits a uniform fiber diameter of ~ 200 nm and a scale-like surface morphology constructed by stacked CNF grains. During scanning, the tip applied a fixed peak force to the CNF surface, allowing precise measurement of its elastic modulus as 4.90 ± 0.75 GPa based on Derjaguin–Muller–Toporov (DMT) model (Fig. 3b). Due to the misaligned assembly of COF grains, the elastic modulus is not homogeneous along the CNF, but varies depending on the specific arrangement of the COF grains (Fig. S19). By screening the elastic modulus of common fibers, the CNF exhibits a similar softness as natural wool while being stiffer than some synthetic nanofibers (Fig. 3c and Table. S4), which makes CNFs unique compared to conventional COF particles in terms of material fabrication and subsequent applications. To visually show the flexibility of the CNFs, we used a focused ion beam (FIB) probe to perform a dynamic bending on the individual CNF (Fig. 3d). By fixing both ends of the CNF and moving the FIB probe, the CNF could be easily bent to a curved state and recover to its original without any cracks (Video S1), highlighting the superior flexibility and mechanical strength of the CNF under the corresponding force.

As illustrated in Fig. 3e, when the CNF is bent, the misaligned COF grains on the upper surface of an individual CNF are squeezed while the grains on the bottom suffer from an extension. This situation could be visually evidenced by examining the SEM images of a bent CNF (inserted graphs in Fig. 3e). On one hand, the misalignment of grains provides sufficient spaces to tolerate such movements. On the other hand, each COF grain contains crystalline/amorphous interfaces, allowing it to be flexible and resistant to external forces at the grain level. Thus, the flexibility of CNF is achieved by a multiscale structural characteristic at both fiber and grain levels. To comprehensively elucidate the flexibility and the mechanical energy dissipation mechanisms in terms of biomimetic scale-like CNFs, a mechanical simulation of the bending process was conducted based on the finite element analysis (FEA). This simulation focused on the examination of the COF grains on the upper and bottom CNF surfaces during bending, while the stress changes are more pronounced on the squeezing side of the bent CNF (Fig. S20). As displayed in Fig. 3f, the deformation of the scale-like CNF on the squeezing side under progressive bending deformation (*ε* = 10% ~ 50%) revealed a three-stage stress evolution: initial localization at intergranular contact zones (*ε* = 10%), followed by planar diffusion along slip planes (*ε* = 30%), and final load redistribution via further planar and inter-grain stress diffusion (*ε* = 50%). The hierarchical stress delocalization mechanism operated through sequential activation of primary stress-transfer networks and secondary load-bearing grains, achieving dual-mechanical energy dissipation via interfacial friction between COF grains and crystalline domain deformation within each grain.

### Processibility and Applications of CNFs

**Fig. 4:**
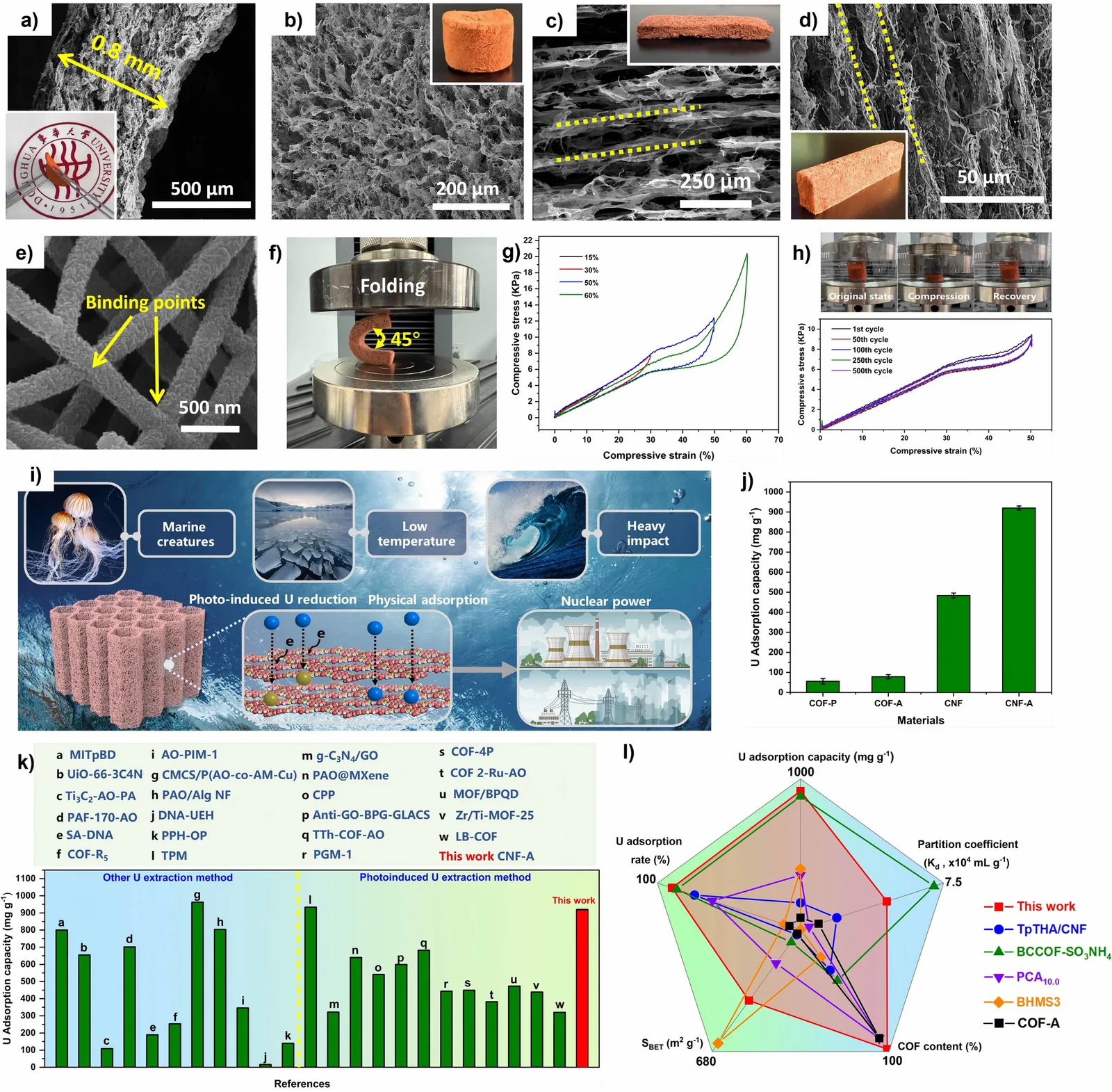
CNF-based materials and their application performances. **a–d** Optical and SEM images of **a** CNF-M, **b** isotropic CNF-A, **c** lamellar CNF-A, and **d** honeycomb CNF-A. **e** Interfibrous binding points in the honeycomb CNF-A formed by photo-induced chemical crosslinking. **f** Photograph of CNF-A under folding. **g** Stress–strain curves of CNF-A under different strains in water. **h** Photograph and stress–strain curves of CNF-A under 500 compression cycles in water. **i** Illustration of CNF-A as an UES material and the harsh service environment it encounters during application. **j** U adsorption capacities of different materials. **k** Comparisons of U adsorption capacity among reported adsorbents. **l** Diamond plot comparing the features of CNF-As with other COF-based aerogels designed for UES

Preparation of pure COF materials with macro-continuity still faces great challenges [35–38]. The direct synthesis of CNFs possessing large *L*/*D* and appropriate fiber length offers a promising strategy to process COFs as common nanofibers into a wide variety of materials with large-scale dimensions and designable microstructures [39–41]. For instance, COF nanofibrous membranes (CNF-Ms) were achieved by vacuum filtration (Figs. 4a and S21). By controlling the concentration of CNFs and the filtration pressure, CNF-Ms could be designed into different thicknesses and densities, fitting to specific application fields. Given the unique bonding point based on physical interweaving and chemical crosslinking applied on the CNF-M, the resultant membrane with a thickness of ~ 0.80 mm exhibits superior flexibility that can be folded and recovered without forming cracks (Fig. S21). More interestingly, nanofibrous aerogels are materials with open-pores in a three-dimensional architecture, which have been widely applied in the fields of energy storage [42–44], pollution management [45–48], sensing [49–51], thermal insulation [52–54], and heterogeneous catalysis [55–57] due to the integrated features of large SSA, high porosity, and lightweight. Although pure COF aerogels have been successfully fabricated by the sol–gel strategy followed by lyophilization, the porous structures and mechanical properties of such aerogels are difficult to be designed and manipulated [15, 58–60]. The direct synthesis of CNFs allows them to be readily homogenized and molded into COF nanofibrous aerogels (CNF-As) with tunable porous topology (including isotropic, lamellar, and honeycomb) and sizes (140 mm × 15 mm (*D* × *H*) and 55 mm × 60 mm (*D* × *H*)) (Fig. S22). The size and shape of the CNF-As could be controlled by varying the size and shape of the mold and the freeze dryer. Their designable porous structures of specific cross section in the CNF-As (labeled in red) are presented under SEM monitoring and shown in Figs. 4b-d and S23–S24. In order to stabilize the structure of as-obtained CNF-As, photo-induced chemical crosslinking was introduced. Specific crosslinking mechanism and parameter selection are available in Figs. S25–29. Considering the mechanical strength, structural stability, and porosity of the CNF-As, the optimal crosslinking parameters were set as MSB concentration of 1.0% and irradiation time of 10 min. Taking honeycomb CNF-A as an example, after UVA irradiation (365 nm, 300 mW cm^−2^), the CNF-A exhibited the optimal mechanical properties with a complete recovery of its shape after compression (Fig. S26). The binding points between CNFs can be observed in Fig. 4e, which enables the structural stability and superelasticity of the aerogels, especially in liquid systems. This interfibrous binding joint was also noticed in other CNF-As (Figs. S23 and 24). First, the structural flexibility of the CNF-A was examined by a folding test. We designed a mold and used it to fabricate a CNF-A with a length of 8.0 cm and a thickness of 1.0 cm. It performs good integrity, and can be folded by 135° and then recovered without structural collapse (Figs. 4f and S30). More importantly, the CNF-A could retain its 3D structure without any release of CNFs after immersing in water for 180 days (Fig. S31). The stress–strain curves of CNF-A in water were acquired at different stains (Fig. 4g), and they exhibit classical closed loops with three characteristic stages: (i) a typical Hookean elastic regime (*ε* < 30%) with a tangential modulus of d*σ*/d*ε* = 26.67 kPa, (ii) a subsequent plateau stage near the yield point (30% < *ε* < 45%), and (iii) a stress hardening region (*ε* > 45%) with stress increasing sharply. Moreover, as displayed in Figs. 4h and S32, the 3D structure of the CNF-A could be completely retained (~ 0% plastic deformation) after 500 compression cycles at *ε* = 50% in water, indicating the superior mechanical stability and superelasticity of the CNF-A (Video S2).

Given the construction of CNF-A by pure COFs, it exhibits comparable and ~ 17 times higher SSA (396.15 m^2^ g^−1^) than that of the CNFs (400.07 m^2^ g^−1^) and COF/nanofiber composite aerogels (COF-As) (23.35 m^2^ g^−1^), respectively (Fig. S33). The COF-As were fabricated by the conventional method of mixing COF particles with supporting nanofibers, which were chosen here as bacterial cellulose nanofibers (see details in the Supplementary Methods and Fig. S34). Additionally, CNFs possess intrinsic photosensitivity by having benzophenone moieties in their chemical skeleton. The engineered hierarchical porosity (Fig. S35) and increased SSA of CNF-As synergistically enhance the photocatalytic efficiency through two mechanisms of increasing active site accessibility and extending light-transporting pathways [61, 62]. In this case, the as-fabricated CNF-As with robust mechanical property and improved photosensitivity could be an optimal prototype for photo-induced uranium extraction from seawater (UES) (Fig. 4i).

Nuclear energy has emerged as a pivotal power source in the near future due to its high energy density and no greenhouse gas emission [63–66]. U is a primary nuclear resource, and its efficient acquisition is essential for the large-scale and sustainable development of the nuclear industry. The U content in seawater exceeds 4.5 billion tons, a thousand times higher than that of terrestrial ore, yet its extraction remains challenging due to the extremely low concentration (~ 3.3 ppb) and competitive ion interference [67–69]. While traditional amidoxime-based adsorbents dominate the current approach, recent advances have shown that photocatalytic UES relying on selective redox conversion achieves superior performance in fibrous adsorbents [2]. The benzophenone moieties in CNFs align their energy bands with the redox potential of U(VI)/U(IV) (Fig. S36), enabling photocatalytic U extraction via the reduction of soluble U(VI) to insoluble U(IV). Detailed mechanism is explained in the Supporting Information. As shown in Fig. 4j and Table S5, the photocatalytic U extraction capacity of CNF-As achieves 920.12 mg g^−1^, which is 16.54 times, 11.72 times and 1.90 times higher than that of the particulate COFs (COF-Ps), COF-As, and CNFs, respectively. It is worth noting that the U extraction by the CNF-A under a dark condition only presents a capacity of 82.25 mg g^−1^, revealing that the photo-induced conversion of U(VI) to U(IV) significantly drives the adsorption reaction (Fig. S37). Moreover, the adsorption kinetics of such materials was evaluated by abstracting the slope from their *Q*_*t*_–*t* curves. As shown in Fig. S38b, CNF-A presents the highest adsorption rate (*k* = 68.39 mg g^−1^ h^−1^) over others. Moreover, the CNF-As performs outstanding U extraction ability compared with numerous reported materials, including particles, membranes, and aerogels based on physical adsorption, photo-induced, and electrical-triggered adsorption mechanisms (Fig. 4k and Table S6) [68, 70–91].

The adsorption selectivity is crucial for extracting U from seawater where abundant competing ions exist. As shown in Fig. S39 and Table S7, CNF-A exhibits a superior adsorption selectivity of U when competed with seven types of metal ions (i.e., V, Fe, Zn, Cu, Ni, Pb, and Co), and the selectivity against *V* reached *U*/*V* = 2.31, making the CNF-A more promising to realize the UES in a practical scenario. The adsorption selectivity of CNF-A is also higher than other control materials, especially for a selectivity coefficient of *U*/*V* = 2.31, which is 2.41, 2.48, and 1.18 times higher than that of COF-P, COF-A, and CNF, respectively (Fig. S39). We also conducted a trial to examine its function in natural seawater. As shown in Fig. S40, the *U* adsorption amount of CNF-A achieved 2.87 mg g^−1^ after 15 days of seawater immersion under light exposure, which is much higher than other typical competing ions, especially for *V* (adsorption amount = 0.74 mg g^−1^), proving CNF-A to have outstanding practical application prospects. During six extraction–elution cycles, the U extraction amount and elution rate only decreased by 1.88% and 2.08% for each cycle, respectively, and finally retained a high adsorption recovery (88.74%) and elution rate (87.54%) (Fig. S41 and Table S8), indicating a superior reusability of the CNF-A. Benefiting from the direct synthesis of CNFs, the as-fabricated CNF-As emerged as one of the most promising COF-based aerogels, demonstrating S_BET_ and COF content outstandingly higher than those of previously reported ones, while performing superior adsorption capacity, adsorption rate, and adsorption selectivity (Fig. 4l and Table S9) [2, 92–94]. In summary, the above results not only emphasized the importance of designing novel adsorbents with 100% COF contents, but also highlighted the advantages of synthesizing COFs into nanofibers and constructing them into 3D aerogels.

## Conclusions

In this study, flexible CNFs with biomimetic scale-like architecture were successfully prepared by the “alcohol-triggered defect cleavage” strategy, which integrated the kinetic control of the reversed Schiff base reaction with the alcohol-mediated crystal reconstruction mechanism. Theoretical simulations were adopted to understand the manipulation function of different alcohols on COF morphology, enabling the direct synthesis of CNFs featuring long length (> 20 μm), record-high *L*/*D* (103.05), and wool-like elastic modulus (~ 4.90 GPa), which overcomes the fundamental limitations of mechanical fragility and processability inherent to conventional COFs. The individual CNF performed superior flexibility and stress resistance, which was attributed to the multiscale stress dispersion among the misaligned COF grains. The further constructed CNF-As exhibited programmable porous structure, high SSA, (396.15 m^2^ g^−1^) and superelasticity (~ 0% structural deformation after 500 compression cycles), and its photo-induced U adsorption capacity (920.12 mg g^−1^) is 11.72 times higher than that of the COF-A, making them promising for practical UES. This work established a facile defect-engineering strategy for constructing mechanically durable COF materials, bridging the long-standing gap between nanoscale precision and macroscopic functionality. The demonstrated synergy between structural engineering and photosensitivity opens new horizons for COFs used in adsorption and separation, heterogeneous catalysis, and energy storage.

## Supplementary Information

Below is the link to the electronic supplementary material.

Supplementary file 1 (DOC 18949 KB)

Supplementary file 2 (MP4 1473 KB)

Supplementary file 3 (MP4 2476 KB)

## References

[1] M. Di, X. Sun, L. Hu, L. Gao, J. Liu et al., Hollow COF selective layer based flexible composite membranes constructed by an integrated “casting-precipitation-evaporation” strategy. Adv. Funct. Mater. **32**(22), 2111594 (2022). 10.1002/adfm.202111594

[2] B. Fan, Y. Si, J. Yu, X. Wang, P. Tang, Multi-interfacial interaction engineered underwater superelastic covalent organic framework aerogels for photoinduced uranium extraction. Chem. Eng. J. **498**, 155756 (2024). 10.1016/j.cej.2024.155756

[3] J. Meng, M. Yin, K. Guo, X. Zhou, Z. Xue, In situ polymerization in COF boosts Li-ion conduction in solid polymer electrolytes for Li metal batteries. Nano-Micro Lett. **17**(1), 248 (2025). 10.1007/s40820-025-01768-3 10.1007/s40820-025-01768-3 PMC12055722 40327199

[4] X. Liu, D. Zhao, J. Wang, Challenges and opportunities in preserving key structural features of 3D-printed metal/covalent organic framework. Nano-Micro Lett. **16**(1), 157 (2024). 10.1007/s40820-024-01373-w 10.1007/s40820-024-01373-w PMC10957829 38512503

[5] Y. Yu, Y. Tang, L. Liu, Q. Wang, C. Yin et al., In situ synthesis of TpPa COFs in mixed matrix membranes for enhanced CO_2_ separation. Ind. Eng. Chem. Res. **64**(16), 8414–8424 (2025). 10.1021/acs.iecr.5c00266

[6] J. Chang, C. Li, X. Wang, D. Li, J. Zhang et al., Quasi-three-dimensional cyclotriphosphazene-based covalent organic framework nanosheet for efficient oxygen reduction. Nano-Micro Lett. **15**(1), 159 (2023). 10.1007/s40820-023-01111-8 10.1007/s40820-023-01111-8 PMC10310679 37386227

[7] C. Xiao, Y. Yao, X. Guo, J. Qi, Z. Zhu et al., Ultralight and robust covalent organic framework fiber aerogels. Small **20**(32), e2311881 (2024). 10.1002/smll.202311881 38372502 10.1002/smll.202311881

[8] G. Yan, X. Sun, Y. Zhang, H. Li, H. Huang et al., Metal-free 2D/2D van der Waals heterojunction based on covalent organic frameworks for highly efficient solar energy catalysis. Nano-Micro Lett. **15**(1), 132 (2023). 10.1007/s40820-023-01100-x 10.1007/s40820-023-01100-x PMC10200743 37211571

[9] X. Xu, Z. Zhang, R. Xiong, G. Lu, J. Zhang et al., Bending resistance covalent organic framework superlattice: “nano-hourglass”-induced charge accumulation for flexible in-plane micro-supercapacitors. Nano-Micro Lett. **15**(1), 25 (2022). 10.1007/s40820-022-00997-0 10.1007/s40820-022-00997-0 PMC9803805 36583830

[10] D. Yan, L. Song, F. Kang, X. Mo, Y. Lv et al., *In situ* growth of covalent organic frameworks on carbon nanotubes for high-performance potassium-ion batteries. Angew. Chem. Int. Ed. **64**(12), e202422851 (2025). 10.1002/anie.202422851 10.1002/anie.202422851 39731475

[11] K. Xu, Y. Zheng, J. Zhou, Y. Zhao, X. Pang et al., Microwave-assisted fabrication of highly crystalline, robust COF membrane for organic solvent nanofiltration. Adv. Funct. Mater. **35**(12), 2417383 (2025). 10.1002/adfm.202417383

[12] F. Yang, J. Guo, C. Han, J. Huang, Z. Zhou et al., Turing covalent organic framework membranes via heterogeneous nucleation synthesis for organic solvent nanofiltration. Sci. Adv. **10**(50), eadr9260 (2024). 10.1126/sciadv.adr9260 39661688 10.1126/sciadv.adr9260 PMC11633759

[13] Y. Kong, X. He, H. Wu, Y. Yang, L. Cao et al., Tight covalent organic framework membranes for efficient anion transport via molecular precursor engineering. Angew. Chem. Int. Ed. **60**(32), 17638–17646 (2021). 10.1002/anie.202105190 10.1002/anie.202105190 34075668

[14] S. Ai, Y. Xu, H. Zhou, Z. Cui, T. Wu et al., Superelastic and ultralight covalent organic framework composite aerogels modified with different functional groups for ultrafast adsorbing organic pollutants in water. Chin. Chem. Lett. **36**(10), 110761 (2025). 10.1016/j.cclet.2024.110761

[15] G. Shao, X. Huang, X. Shen, C. Li, A. Thomas, Metal-organic framework and covalent-organic framework-based aerogels: synthesis, functionality, and applications. Adv. Sci. **11**(48), 2409290 (2024). 10.1002/advs.202409290 10.1002/advs.202409290 PMC11672323 39467257

[16] J.Á. Martín-Illán, J.A. Suárez, J. Gómez-Herrero, P. Ares, D. Gallego-Fuente et al., Ultralarge free-standing imine-based covalent organic framework membranes fabricated via compression. Adv. Sci. **9**(7), 2104643 (2022). 10.1002/advs.202104643 10.1002/advs.202104643 PMC8895050 35038248

[17] Y. Zhang, S. Liu, J. Yan, X. Zhang, S. Xia et al., Superior flexibility in oxide ceramic crystal nanofibers. Adv. Mater. **33**(44), 2105011 (2021). 10.1002/adma.202105011 10.1002/adma.202105011 34532907

[18] X. Ma, K.R. Meihaus, Y. Yang, Y. Zheng, F. Cui et al., Photocatalytic extraction of uranium from seawater using covalent organic framework nanowires. J. Am. Chem. Soc. **146**(33), 23566–23573 (2024). 10.1021/jacs.4c07699 39121013 10.1021/jacs.4c07699

[19] H. Yang, L. Yang, H. Wang, Z. Xu, Y. Zhao et al., Covalent organic framework membranes through a mixed-dimensional assembly for molecular separations. Nat. Commun. **10**(1), 2101 (2019). 10.1038/s41467-019-10157-5 31068595 10.1038/s41467-019-10157-5 PMC6506600

[20] S. Wang, Z. Zhang, H. Zhang, A.G. Rajan, N. Xu et al., Reversible polycondensation-termination growth of covalent-organic-framework spheres, fibers, and films. Matter **1**(6), 1592–1605 (2019). 10.1016/j.matt.2019.08.019

[21] S. Daliran, A.R. Oveisi, A. Dhakshinamoorthy, H. Garcia, Probing defects in covalent organic frameworks. ACS Appl. Mater. Interfaces **16**(38), 50096–50114 (2024). 10.1021/acsami.4c12069 39283167 10.1021/acsami.4c12069 PMC12557228

[22] K. Du, L. Xiong, C. Fu, X. Ni, J.-L. Bredas et al., Impact of structural defects on the electronic properties of two-dimensional covalent organic frameworks. ACS Mater. Lett. **6**(2), 335–344 (2024). 10.1021/acsmaterialslett.3c01429

[23] S. Daliran, M. Blanco, A. Dhakshinamoorthy, A.R. Oveisi, J. Alemán et al., Defects and disorder in covalent organic frameworks for advanced applications. Adv. Funct. Mater. **34**(18), 2312912 (2024). 10.1002/adfm.202312912

[24] Z. Li, Z.-W. Liu, Z. Li, T.-X. Wang, F. Zhao et al., Defective 2D covalent organic frameworks for postfunctionalization. Adv. Funct. Mater. **30**(10), 1909267 (2020). 10.1002/adfm.201909267

[25] Z. Guo, H. Wu, Y. Chen, S. Zhu, H. Jiang et al., Missing-linker defects in covalent organic framework membranes for efficient CO_2_ separation. Angew. Chem. Int. Ed. **61**(41), e202210466 (2022). 10.1002/anie.202210466 10.1002/anie.202210466 35980347

[26] T. Ma, E.A. Kapustin, S.X. Yin, L. Liang, Z. Zhou et al., Single-crystal X-ray diffraction structures of covalent organic frameworks. Science **361**(6397), 48–52 (2018). 10.1126/science.aat7679 29976818 10.1126/science.aat7679

[27] J. Zhang, Z. Wang, J. Suo, C. Tuo, F. Chen et al., Morphological tuning of covalent organic framework single crystals. J. Am. Chem. Soc. **146**(51), 35090–35097 (2024). 10.1021/jacs.4c10071 39670337 10.1021/jacs.4c10071

[28] G. Zhan, B. Koek, Y. Yuan, Y. Liu, V. Mishra et al., Moiré two-dimensional covalent organic framework superlattices. Nat. Chem. **17**(4), 518–524 (2025). 10.1038/s41557-025-01748-5 39979413 10.1038/s41557-025-01748-5 PMC12095054

[29] S. Chen, D.M. Koshy, Y. Tsao, R. Pfattner, X. Yan et al., Highly tunable and facile synthesis of uniform carbon flower particles. J. Am. Chem. Soc. **140**(32), 10297–10304 (2018). 10.1021/jacs.8b05825 30067349 10.1021/jacs.8b05825

[30] S.-A. Chen, Polymer miscibility in organic solvents and in plasticizers: a two-dimensional approach. J. Appl. Polym. Sci. **15**(5), 1247–1266 (1971). 10.1002/app.1971.070150519

[31] C. Lefebvre, G. Rubez, H. Khartabil, J.-C. Boisson, J. Contreras-García et al., Accurately extracting the signature of intermolecular interactions present in the NCI plot of the reduced density gradient *versus* electron density. Phys. Chem. Chem. Phys. **19**(27), 17928–17936 (2017). 10.1039/C7CP02110K 28664951 10.1039/c7cp02110k

[32] Z. Yan, X. Liu, B. Ding, J. Yu, Y. Si, Interfacial engineered superelastic metal-organic framework aerogels with van-der-Waals barrier channels for nerve agents decomposition. Nat. Commun. **14**(1), 2116 (2023). 10.1038/s41467-023-37693-5 37055384 10.1038/s41467-023-37693-5 PMC10101950

[33] Z. Zhang, B. Yang, B. Zhang, M. Cui, J. Tang et al., Type II porous ionic liquid based on metal-organic cages that enables L-tryptophan identification. Nat. Commun. **13**(1), 2353 (2022). 10.1038/s41467-022-30092-2 35487897 10.1038/s41467-022-30092-2 PMC9054828

[34] G.J. Fan, H. Choo, P.K. Liaw, E.J. Lavernia, A model for the inverse Hall–Petch relation of nanocrystalline materials. Mater. Sci. Eng. A **409**(1–2), 243–248 (2005). 10.1016/j.msea.2005.06.073

[35] Q. Li, Y. Zhu, T. Pan, G. Zhang, H. Pang, Covalent organic framework nanomaterials: syntheses, architectures, and applications. Adv. Colloid Interface Sci. **339**, 103427 (2025). 10.1016/j.cis.2025.103427 39929054 10.1016/j.cis.2025.103427

[36] K. Liu, C. Yin, J. Gao, Y. Wang, Temperature-swing synthesis of highly crystalline covalent organic framework films for fast and precise molecular separations. Angew. Chem. Int. Ed. **64**(12), e202422333 (2025). 10.1002/anie.202422333 10.1002/anie.202422333 39673084

[37] S. Kandambeth, K. Dey, R. Banerjee, Covalent organic frameworks: chemistry beyond the structure. J. Am. Chem. Soc. **141**(5), 1807–1822 (2019). 10.1021/jacs.8b10334 30485740 10.1021/jacs.8b10334

[38] C. Fan, H. Wu, J. Guan, X. You, C. Yang et al., Scalable fabrication of crystalline COF membranes from amorphous polymeric membranes. Angew. Chem. Int. Ed. **60**(33), 18051–18058 (2021). 10.1002/anie.202102965 10.1002/anie.202102965 34062042

[39] P.N. Anjana, A.K. Pulikkal, Synthesis, derivation, and applications of imine-linked covalent organic frameworks: a comprehensive review. Microporous Mesoporous Mater. **387**, 113516 (2025). 10.1016/j.micromeso.2025.113516

[40] S. Xiong, Y. Wang, X. Wang, J. Chu, R. Zhang et al., Schiff base type conjugated organic framework nanofibers: solvothermal synthesis and electrochromic properties. Sol. Energy Mater. Sol. Cells **209**, 110438 (2020). 10.1016/j.solmat.2020.110438

[41] X. Wang, H. Liu, S. Chen, J. Zhang, S. Chen, *In situ* construction of covalent organic framework membranes on polyacrylonitrile nanofibers for carbon dioxide capture. ACS Appl. Nano Mater. **7**(9), 10911–10920 (2024). 10.1021/acsanm.4c01174

[42] M. Zhang, Y. Wang, Y. Zhang, J. Song, Y. Si et al., Conductive and elastic TiO_2_ nanofibrous aerogels: a new concept toward self-supported electrocatalysts with superior activity and durability. Angew. Chem. Int. Ed. **59**(51), 23252–23260 (2020). 10.1002/anie.202010110 10.1002/anie.202010110 32881302

[43] B. Ding, S. Huang, K. Pang, Y. Duan, J. Zhang, Nitrogen-enriched carbon nanofiber aerogels derived from marine chitin for energy storage and environmental remediation. ACS Sustainable Chem. Eng. **6**(1), 177–185 (2018). 10.1021/acssuschemeng.7b02164

[44] M. Cho, J. Yiu, L.-T. Lin, Q. Hua, M.A. Karaaslan et al., Lignin nanofiber flexible carbon aerogels for self-standing supercapacitors. Chemsuschem **18**(3), e202400932 (2025). 10.1002/cssc.202400932 39304517 10.1002/cssc.202400932 PMC11789977

[45] W.M. Saleh, M.I. Ahmad, E.B. Yahya, H.P.S. Abdul Khalil, Nanostructured bioaerogels as a potential solution for particulate matter pollution. Gels **9**(7), 575 (2023). 10.3390/gels9070575 37504454 10.3390/gels9070575 PMC10379271

[46] X. Li, X. Ba, Y. Dai, Y. Feng, S. Yan et al., Silk nanofibrillar aerogel as sustainable filters for environmental purification. Small **21**(12), e2500226 (2025). 10.1002/smll.202500226 39955764 10.1002/smll.202500226

[47] F. Wang, J. Dai, L. Huang, Y. Si, J. Yu et al., Biomimetic and superelastic silica nanofibrous aerogels with rechargeable bactericidal function for antifouling water disinfection. ACS Nano **14**(7), 8975–8984 (2020). 10.1021/acsnano.0c03793 32644778 10.1021/acsnano.0c03793

[48] P. Tang, B. Fan, Y. Wang, Y. Si, J. Yu et al., Interfacial engineered, hierarchically porous, and underwater superelastic nanofibrous aerogels with rime-mimetic structure for superior micropollutant extraction. Chem. Eng. J. **475**, 146290 (2023). 10.1016/j.cej.2023.146290

[49] J. Lin, J. Li, Y. Song, W. Chu, W. Li et al., Carbon nanofibrous aerogels derived from electrospun polyimide for multifunctional piezoresistive sensors. ACS Appl. Mater. Interfaces **16**(13), 16712–16723 (2024). 10.1021/acsami.4c00452 38506548 10.1021/acsami.4c00452

[50] J. Lin, J. Li, W. Li, S. Chen, Y. Lu et al., Multifunctional polyimide nanofibrous aerogel sensor for motion monitoring and airflow perception. Compos. Part A Appl. Sci. Manuf. **178**, 108003 (2024). 10.1016/j.compositesa.2023.108003

[51] Y. Si, J. Yu, X. Tang, J. Ge, B. Ding, Ultralight nanofibre-assembled cellular aerogels with superelasticity and multifunctionality. Nat. Commun. **5**, 5802 (2014). 10.1038/ncomms6802 25512095 10.1038/ncomms6802

[52] J. Guo, S. Fu, Y. Deng, X. Xu, S. Laima et al., Hypocrystalline ceramic aerogels for thermal insulation at extreme conditions. Nature **606**(7916), 909–916 (2022). 10.1038/s41586-022-04784-0 35768591 10.1038/s41586-022-04784-0 PMC9242853

[53] J. Li, H. Li, J. Lin, Y. Lu, J. Qin et al., Multilayer polyimide nanofibrous aerogels for efficient thermal insulation and piezoelectric sensor. Chem. Eng. J. **507**, 160807 (2025). 10.1016/j.cej.2025.160807

[54] X. Fu, L. Si, Z. Zhang, T. Yang, Q. Feng et al., Gradient all-nanostructured aerogel fibers for enhanced thermal insulation and mechanical properties. Nat. Commun. **16**(1), 2357 (2025). 10.1038/s41467-025-57646-4 40064924 10.1038/s41467-025-57646-4 PMC11893758

[55] J. Qiu, W. Zheng, R. Yuan, C. Yue, D. Li et al., A novel 3D nanofibrous aerogel-based MoS_2_@Co_3_S_4_ heterojunction photocatalyst for water remediation and hydrogen evolution under simulated solar irradiation. Appl. Catal. B Environ. **264**, 118514 (2020). 10.1016/j.apcatb.2019.118514

[56] G. Jiang, J. Wang, Y. Song, W. Chen, Y. Ye et al., Facile synthesis of ZIF-67@PVA/CA nanofibrous aerogel as efficient and recyclable catalyst for the degradation of organic pollutants through peroxymonosulfate activation. J. Appl. Polym. Sci. **140**(28), e54033 (2023). 10.1002/app.54033

[57] D.B. Jung, Y. Song, Y.-R. Lee, M.J. Cha, K. Jeong et al., Quaternarized chitosan nanofiber and ZIF aerogel composites for synergetic CO_2_ cycloaddition catalysis. Carbohydr. Polym. **347**, 122685 (2025). 10.1016/j.carbpol.2024.122685 39486928 10.1016/j.carbpol.2024.122685

[58] D. Zhu, Y. Zhu, Q. Yan, M. Barnes, F. Liu et al., Pure crystalline covalent organic framework aerogels. Chem. Mater. **33**(11), 4216–4224 (2021). 10.1021/acs.chemmater.1c01122

[59] Z. Sheng, Z. Liu, Y. Hou, H. Jiang, Y. Li et al., The rising aerogel fibers: status, challenges, and opportunities. Adv. Sci. **10**(9), 2205762 (2023). 10.1002/advs.202205762 10.1002/advs.202205762 PMC10037991 36658735

[60] Q. Wang, P. Wang, Y. Wang, Y. Xu, H. Xu et al., Design of high-performance formyl-functionalized COF aerogels as quasi-solid lithium battery electrolyte by a solvent substitution strategy. ACS Appl. Mater. Interfaces **16**(28), 37052–37062 (2024). 10.1021/acsami.4c07017 38965714 10.1021/acsami.4c07017

[61] W. Chi, Y. Dong, B. Liu, C. Pan, J. Zhang et al., A photocatalytic redox cycle over a polyimide catalyst drives efficient solar-to-H_2_O_2_ conversion. Nat. Commun. **15**(1), 5316 (2024). 10.1038/s41467-024-49663-6 38909037 10.1038/s41467-024-49663-6 PMC11535368

[62] M. Zhang, M. Lu, Z.-L. Lang, J. Liu, M. Liu et al., Semiconductor/covalent-organic-framework Z-scheme heterojunctions for artificial photosynthesis. Angew. Chem. Int. Ed. **59**(16), 6500–6506 (2020). 10.1002/anie.202000929 10.1002/anie.202000929 31989745

[63] J.H.C. Ng, P. Vyawahare, P.T. Benavides, Y. Gan, P. Sun et al., Life-cycle greenhouse gas emissions associated with nuclear power generation in the United States. J. Ind. Ecol. **29**(3), 719–732 (2025). 10.1111/jiec.70008

[64] F. Pomponi, J. Hart, The greenhouse gas emissions of nuclear energy–life cycle assessment of a European pressurised reactor. Appl. Energy **290**, 116743 (2021). 10.1016/j.apenergy.2021.116743

[65] M.D. Mathew, Nuclear energy: a pathway towards mitigation of global warming. Prog. Nucl. Energy **143**, 104080 (2022). 10.1016/j.pnucene.2021.104080

[66] M.Y. Mehboob, B. Ma, M. Sadiq, Y. Zhang, Does nuclear energy reduce consumption-based carbon emissions: the role of environmental taxes and trade globalization in highest carbon emitting countries. Nucl. Eng. Technol. **56**(1), 180–188 (2024). 10.1016/j.net.2023.09.022

[67] D. Zhang, L. Fang, L. Liu, B. Zhao, B. Hu et al., Uranium extraction from seawater by novel materials: a review. Sep. Purif. Technol. **320**, 124204 (2023). 10.1016/j.seppur.2023.124204

[68] Y. Yuan, Q. Yu, M. Cao, L. Feng, S. Feng et al., Selective extraction of uranium from seawater with biofouling-resistant polymeric peptide. Nat. Sustain. **4**(8), 708–714 (2021). 10.1038/s41893-021-00709-3

[69] D. Chen, X. Zhao, M. Shi, X. Fu, W. Hu et al., Enhanced and selective uranium extraction onto electrospun nanofibers by regulating the functional groups and photothermal conversion performance. Chem. Eng. J. **480**, 148108 (2024). 10.1016/j.cej.2023.148108

[70] W. Zhang, M. Wu, Y. Xin, H. Liu, F. Li et al., Comparative analysis of seawater uranium extraction materials: toward the development of bio-based and biomimetic materials. Coord. Chem. Rev. **534**, 216589 (2025). 10.1016/j.ccr.2025.216589

[71] Y. Yuan, S. Feng, L. Feng, Q. Yu, T. Liu et al., A bio-inspired nano-pocket spatial structure for targeting uranyl capture. Angew. Chem. Int. Ed. **59**(11), 4262–4268 (2020). 10.1002/anie.201916450 10.1002/anie.201916450 31908089

[72] D. Zhang, L. Liu, B. Zhao, X. Wang, H. Pang et al., Highly efficient extraction of uranium from seawater by polyamide and amidoxime co-functionalized MXene. Environ. Pollut. **317**, 120826 (2023). 10.1016/j.envpol.2022.120826 36493939 10.1016/j.envpol.2022.120826

[73] Z. Li, Q. Meng, Y. Yang, X. Zou, Y. Yuan et al., Constructing amidoxime-modified porous adsorbents with open architecture for cost-effective and efficient uranium extraction. Chem. Sci. **11**(18), 4747–4752 (2020). 10.1039/d0sc00249f 34122930 10.1039/d0sc00249f PMC8159166

[74] W. Zhang, Y. Xin, Y. Fa, F. Li, Y. Liu et al., SA-DNA hydrogel microspheres for ultra-selective uranyl (VI) extraction from seawater. Chem. Eng. J. **495**, 153690 (2024). 10.1016/j.cej.2024.153690

[75] Y. Xie, Y. Wu, X. Liu, M. Hao, Z. Chen et al., Rational design of cooperative chelating sites on covalent organic frameworks for highly selective uranium extraction from seawater. Cell Rep. Phys. Sci. **4**(1), 101220 (2023). 10.1016/j.xcrp.2022.101220

[76] Y. Zhang, Y. Wang, Z. Dong, Y. Wang, Y. Liu et al., Boosting uranium extraction from seawater by micro-redox reactors anchored in a seaweed-like adsorbent. Nat. Commun. **15**(1), 9124 (2024). 10.1038/s41467-024-53366-3 39443537 10.1038/s41467-024-53366-3 PMC11500014

[77] X. Xu, Y. Yue, D. Cai, J. Song, C. Han et al., Aqueous solution blow spinning of seawater-stable polyamidoxime nanofibers from water-soluble precursor for uranium extraction from seawater. Small Methods **4**(12), 2000558 (2020). 10.1002/smtd.202000558

[78] L. Yang, H. Xiao, Y. Qian, X. Zhao, X.-Y. Kong et al., Bioinspired hierarchical porous membrane for efficient uranium extraction from seawater. Nat. Sustain. **5**(1), 71–80 (2022). 10.1038/s41893-021-00792-6

[79] Y. Yuan, T. Liu, J. Xiao, Q. Yu, L. Feng et al., DNA nano-pocket for ultra-selective uranyl extraction from seawater. Nat. Commun. **11**(1), 5708 (2020). 10.1038/s41467-020-19419-z 33177515 10.1038/s41467-020-19419-z PMC7659010

[80] B. Zhang, X. Shan, J. Yu, H. Zhang, K.T. Alali et al., Facile synthesis of TiO_2_-PAN photocatalytic membrane with excellent photocatalytic performance for uranium extraction from seawater. Sep. Purif. Technol. **328**, 125026 (2024). 10.1016/j.seppur.2023.125026

[81] Y. Liao, B. Yuan, D. Zhang, J. Zhang, X. Wang et al., Fabrication of heterostructured metal oxide/TiO_2_ nanotube arrays prepared via thermal decomposition and crystallization. Inorg. Chem. **57**(16), 10249–10256 (2018). 10.1021/acs.inorgchem.8b01483 30074777 10.1021/acs.inorgchem.8b01483

[82] M. Fu, C. Huang, L. Ma, Y. Yao, J. Chen et al., Solar enhanced uranium extraction from seawater with the efficient strategy of MXene loaded nano-porous polyamidoxime membrane. Sep. Purif. Technol. **332**, 125803 (2024). 10.1016/j.seppur.2023.125803

[83] W. Cui, C. Zhang, R. Liang, J. Qiu, Covalent organic framework hydrogels for synergistic seawater desalination and uranium extraction. J. Mater. Chem. A **9**(45), 25611–25620 (2021). 10.1039/D1TA06732J

[84] T. Li, X. Lin, Z. Zhang, L. Yang, Y. Qian et al., Photothermal-enhanced uranium extraction from seawater: a biomass solar thermal collector with 3D ion-transport networks. Adv. Funct. Mater. **33**(19), 2212819 (2023). 10.1002/adfm.202212819

[85] F. Yu, C. Li, W. Li, Z. Yu, Z. Xu et al., Π-skeleton tailoring of olefin-linked covalent organic frameworks achieving low exciton binding energy for photo-enhanced uranium extraction from seawater. Adv. Funct. Mater. **34**(1), 2307230 (2024). 10.1002/adfm.202307230

[86] Z. Liu, K. Feng, X. Zhang, L. Fu, J. Ren et al., Enhanced uranium extraction using a nanostructured photothermal hydrogel membrane. Chem. Eng. J. **498**, 155423 (2024). 10.1016/j.cej.2024.155423

[87] H. Yang, M. Hao, Y. Xie, X. Liu, Y. Liu et al., Tuning local charge distribution in multicomponent covalent organic frameworks for dramatically enhanced photocatalytic uranium extraction. Angew. Chem. Int. Ed. **62**(30), e202303129 (2023). 10.1002/anie.202303129 10.1002/anie.202303129 37117155

[88] M. Hao, Y. Xie, X. Liu, Z. Chen, H. Yang et al., Modulating uranium extraction performance of multivariate covalent organic frameworks through donor–acceptor linkers and amidoxime nanotraps. JACS Au **3**(1), 239–251 (2023). 10.1021/jacsau.2c00614 36711090 10.1021/jacsau.2c00614 PMC9875373

[89] M. Chen, T. Liu, X. Zhang, R. Zhang, S. Tang et al., Photoinduced enhancement of uranium extraction from seawater by MOF/black phosphorus quantum dots heterojunction anchored on cellulose nanofiber aerogel. Adv. Funct. Mater. **31**(22), 2100106 (2021). 10.1002/adfm.202100106

[90] T. Liu, S. Tang, T. Wei, M. Chen, Z. Xie et al., Defect-engineered metal-organic framework with enhanced photoreduction activity toward uranium extraction from seawater. Cell Rep. Phys. Sci. **3**(5), 100892 (2022). 10.1016/j.xcrp.2022.100892

[91] Y. Zhao, S. Li, G. Fu, H. Yang, S. Li et al., Construction of layer-blocked covalent organic framework heterogenous films via surface-initiated polycondensations with strongly enhanced photocatalytic properties. ACS Cent. Sci. **10**(4), 775–781 (2024). 10.1021/acscentsci.3c01195 38680569 10.1021/acscentsci.3c01195 PMC11046463

[92] M. Li, B. Qing, H. Luo, W. Gao, Q. Shou et al., Recyclable covalent organic frameworks/cellulose aerogels for efficient uranium adsorption. Int. J. Biol. Macromol. **282**(Pt 4), 137156 (2024). 10.1016/j.ijbiomac.2024.137156 39488314 10.1016/j.ijbiomac.2024.137156

[93] M. Li, L. Sun, W. Gao, B. Qing, H. Yao et al., *In-situ* bioprocessing of bacterial cellulose aerogel with covalent organic frameworks for enhanced uranium extraction. Sep. Purif. Technol. **355**, 129654 (2025). 10.1016/j.seppur.2024.129654

[94] W. Cui, C. Zhang, R. Liang, J. Liu, J. Qiu, Covalent organic framework sponges for efficient solar desalination and selective uranium recovery. ACS Appl. Mater. Interfaces **13**(27), 31561–31568 (2021). 10.1021/acsami.1c04419 34192870 10.1021/acsami.1c04419

