# Supplementary material for: Flexible High-Aspect-Ratio COF Nanofibers: Defect-Engineered Synthesis, Superelastic Aerogels, and Uranium Extraction Applications
Source: Nanomicro Lett. 2026 Jan 12;18:142. doi: 10.1007/s40820-025-01984-x (PMC12791102; doi:10.1007/s40820-025-01984-x)
Supplement: Supplementary file 1 — (DOC 18949 KB) [file 40820_2025_1984_MOESM1_ESM.doc]

Supporting Information for

**Flexible High-Aspect-Ratio COF Nanofibers: Defect-Engineered Synthesis, Superelastic Aerogels, and Uranium Extraction Applications**

Binbin Fan^1^, Jianyong Yu^2^, Xueli Wang^2^ *, Yang Si^2^ *, Peixin Tang^2^ *

^1^ College of Textiles, Donghua University, Shanghai 201620, P. R. China

^2^ Innovation Center for Textile Science and Technology, Donghua University, Shanghai 201620, P. R. China

*Corresponding authors. E-mail: [wxl@dhu.edu.cn](mailto:wxl@dhu.edu.cn) (Xueli Wang); [yangsi@dhu.edu.cn](mailto:yangsi@dhu.edu.cn) (Yang Si); [pxtang@dhu.edu.cn](mailto:pxtang@dhu.edu.cn) (Peixin Tang)

**S1 Supplementary Methods**

**Materials.** Uranyl nitrate hexahydrate (99%, MACKLIN), bacterial cellulose nanofibers dispersion (BCN, 0.8 wt%, Qihong, Guilin, Guangxi, China), sodium hydroxide (96%, Collins), hydrochloric acid (CP, SCR), uranium standard solution (Beijing Century Oko Biotechnology Co., Ltd., China), cupric chloride anhydrous (CuCl_2_, 98%, Adamas-bata), zinc nitrate hexahydrate (Zn(NO_3_)_2_·6H_2_O, AR, SCR), iron (III) nitrate nonahydrate (Fe(NO_3_)_3_·9H_2_O, 98.5%, MACKLIN), nickel (II) acetate tetrahydrate (Ni(OCOCH_3_)_2_·4H_2_O, 99%, Infinity Scientific), ammonium metavanadate (NH_4_VO_3_, 99%, MERYER), cobaltous nitrate hexahydrate (Co(NO_3_)_2_·6H_2_O, 99%, RHAWN), lead(II) nitrate (Pb(NO_3_)_2_, 99%, AR, SCR). All chemicals are directly used as received.

**Characterizations.** Attenuated total reflection spectroscopy (ATR) was acquired by a Nicolet TM iS50 spectrometer (Thermo Fisher Scientific). The transmittance mode was used, and the data were collected in the wavenumbers of 400~4000 cm^-1^ with a resolution of 2 cm^-1^. ^13^C SSNMR was acquired from a Bruker AVANCE NEO 400M (German) spectrometer utilizing TMS (0 ppm) as a reference, and the spectra were processed using the MestReNova software. Atomic force microscopy (AFM) was performed on Bruker Dimension Icon (German), and all the original data were processed using the NanoScope Analysis 3.00 software. Thermogravimetric analysis (TGA) was performed on a TGA2 (Mettler-Toledo) instrument. Firstly, the sample (~5 mg) was heated to 100^o^C and held for 1 min to remove any free water. Then, the system was cooled to room temperature under N_2_ atmosphere and reheated to 700 ^o^C with a heating rate and N_2_ flow rate of 20^o^C min^-1^ and 50 mL min^-1^, respectively. The samples were coated with a thin layer of gold before scanning electron microscope (SEM) monitoring. The SEM images of the materials were captured on an SU8010 (Hitachi, Japan). Transmission electron microscope (TEM, JEM-2100F) was operated at a voltage of 200 kV. X-ray diffraction (XRD) was acquired by SmartLab SE (Rigaku, Japan). The sample was scanned at a scanning rate of 3^o^ min^-1^ from 3^o^ to 80^o^ with a test target of cuprum. N_2_ sorption isotherm was obtained from a physisorption system of BSD-660 (Beishide Instrument-S&T. (Beijing) Co., Ltd.) at 77 K. The samples were degassed at 100ºC for 5h before testing. The BET surface areas were acquired based on BET theory. The pore volumes were obtained according to the Barrett-Joyner-Halenda (BJH) model. An ultraviolet-visible spectrophotometer (UV-Vis) of UV3600 (SHIMADZU, Japan) was used to collect the adsorption spectra of materials in the wavelengths of 200~1000 nm. All the light sources are standard (Halogen Tungsten Lamp (Model 64604), Deuterium lamp (Model L6380)) and the lights are in their working life-span (<2000 h). Inductively coupled plasma emission spectrometer-atomic emission spectrometer (ICP-OES) was performed on Prodigy Plus (Teledyne Leeman Labs, USA).

**Fabrication of COF-A.** BCNs and COF particles were homogenized under ultrasonication for 10 min. Then, the suspension of BCN/COF particle was shaped into nanofibrous aerogels (COF-As) via liquid N_2_ freezing and lyophilization. Citric acid (CA) was used as the crosslinking agent for enhancing the structural stability of aerogels. By heating the aerogels under 160^o^C for 30 min, esterification reactions occurred between CA and BCNs, thus inhibiting the re-dispersion of BCNs in water during practical applications. Given the combined interaction of hydrogen bonding and ester bond crosslinking, COF-A with 90.91wt% COF loading was fabricated.

**Compression Tests of CNF-A.** The compression tests were performed on an Exceed Model E42 (MTS Systems Co., Shanghai, China) with a constant compression rate of 60.00 mm min^-1^. The samples for compression tests were in a cylinder shape with a diameter of 2 cm and a height of 2.5 cm. The compression cycle test was performed for 500 cycles in distilled water at a maximum strain of 50%.

**Adsorption of CNF-A.** The U adsorption performance was first conducted by dissolving a specific amount of uranyl nitrate (UO_2_(NO_3_)_2_) in distilled water (initial concentration=128 ppm, pH=4). In each specific adsorption test, 25 mg of the adsorbent was added to 200 mL of U-spiked distilled water. Then, the reaction systems were irradiated by visible light (405 nm, 300 mW cm^-2^) in a crosslinking box (UVGO, China). The distance between sample and lamps was controlled as 8.0 cm. Adsorption selectivity examination was performed in metal ion-spiked distilled water. UO_2_^2+^ and competitive metal ions (V^5+^, Fe^3+^, Zn^2+^, Cu^2+^, Ni^2+^, Pb^2+^, Co^2+^), whose concentrations are 100 times higher than their actual concentration in natural seawater, were dissolved in distilled water. Then, 100 mg of CNF-A adsorbent was added to the mixture solution (1.0 L) to perform the competitive adsorption assay. After 24 h of the adsorption under visible light irradiation, the CNF-A was taken out and then dried at 60℃ under a vacuum for 12 h. Additionally, the U adsorption performance in natural seawater (5.0 L) was conducted using 5 mg of CNF-A adsorbent. The natural seawater was collected from Laoshan (Qingdao, China). After 24 h of the adsorption under visible light irradiation, the CNF-A adsorbent was taken out and then dried at 60℃ under vacuum for 12 h. The mass content of the adsorbed metal ions in CNF-A was analyzed by ICP-OES after microwave digestion.

**Computational Simulations.** Calculations including ground state structure optimization and frequency calculations based on density functional theory (DFT) were performed with Gaussian 09 at unrestricted DFT-B3LYP/6-31G(d,p), nosymm level of theory under gas phase (i.e., no solvent system model was applied). No imaginary frequency was found in the frequency calculation results, indicating all the optimized geometries are at their minimal energy states. The optimized geometries of the interested compounds and composites were obtained and viewed by GaussView 5.0.

**S2 Supplementary Figures**

**
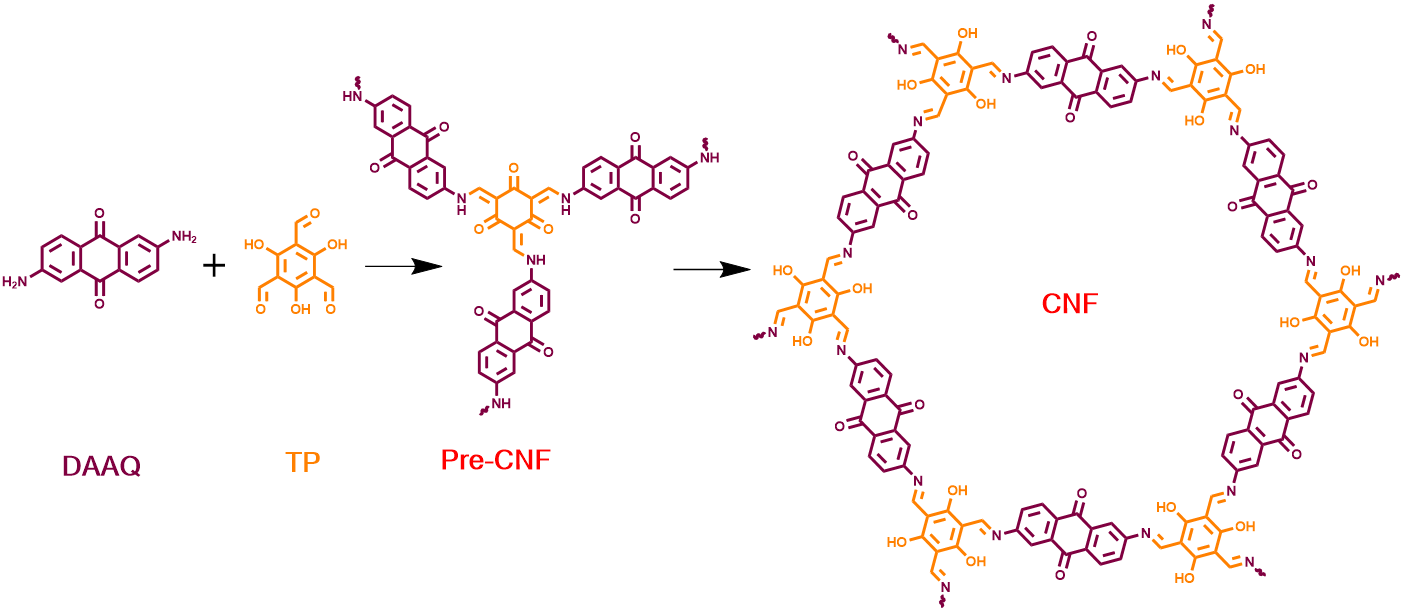
**

**Fig. S1** Synthesis reaction and chemical structure of CNF synthesized by DAAQ and TP


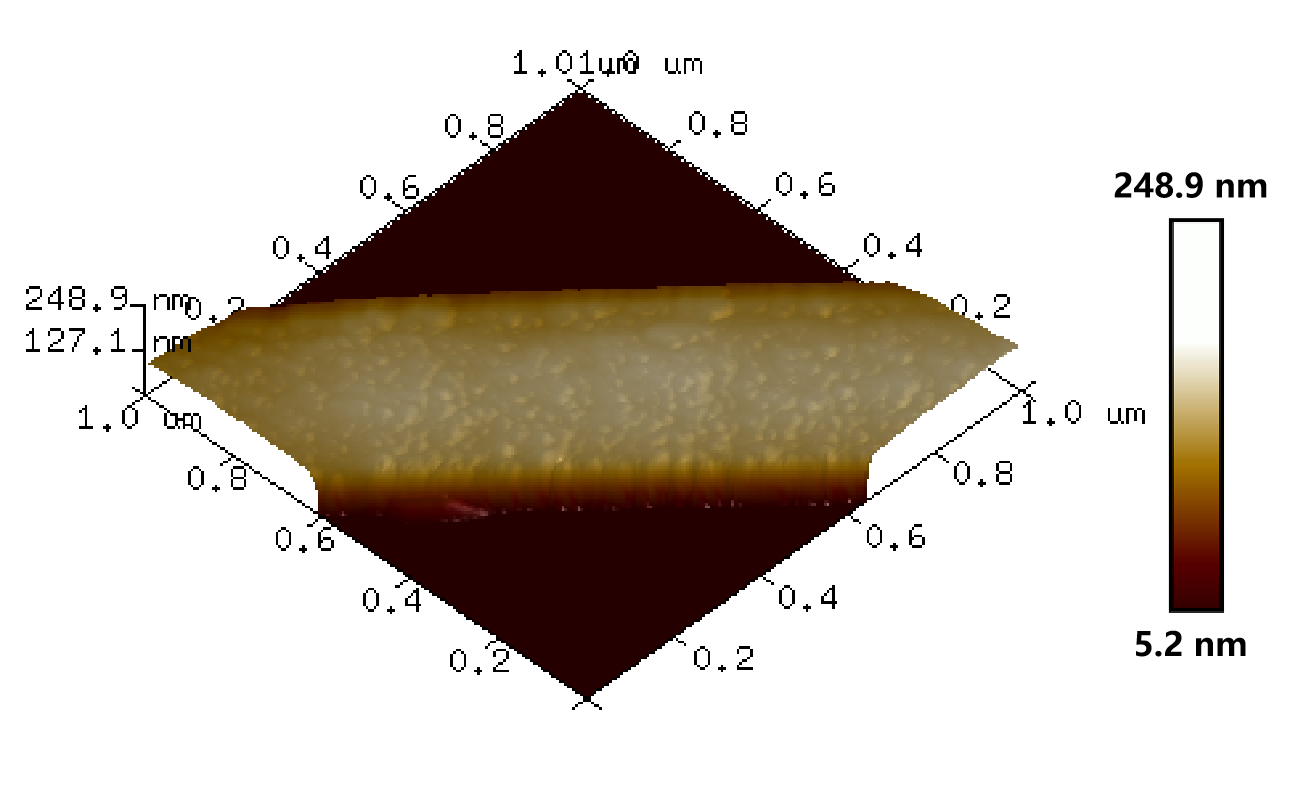


**Fig. S2** AFM image of an individual CNF


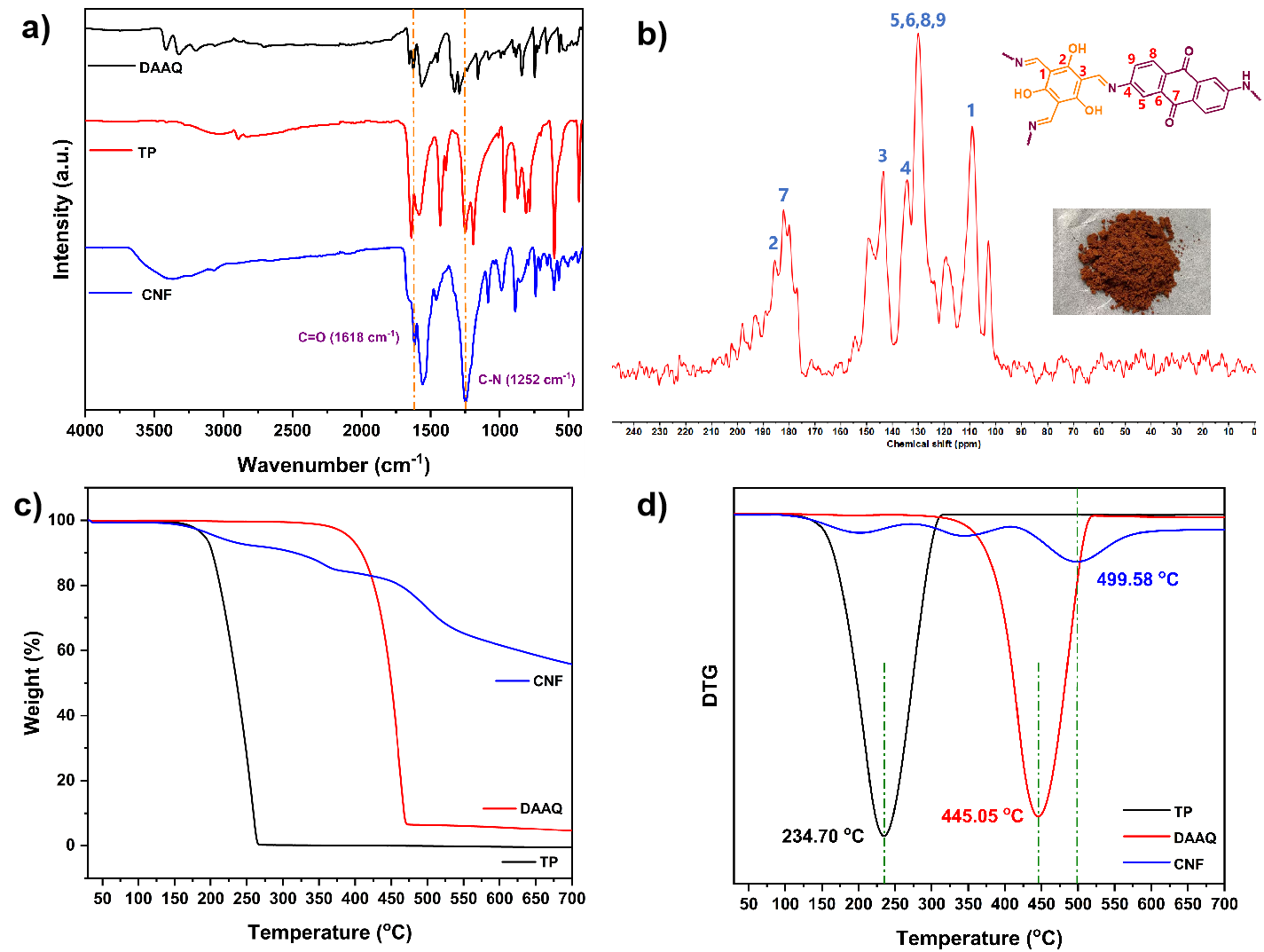


**Fig. S3 a** ATR-FTIR spectra of TP, DAAQ and CNF. **b** ^13^C SSNMR of CNF. The inserted photograph shows the optical image of CNFs. **c** The TGA and **d** DTG curves of TP, DAAQ, and CNF


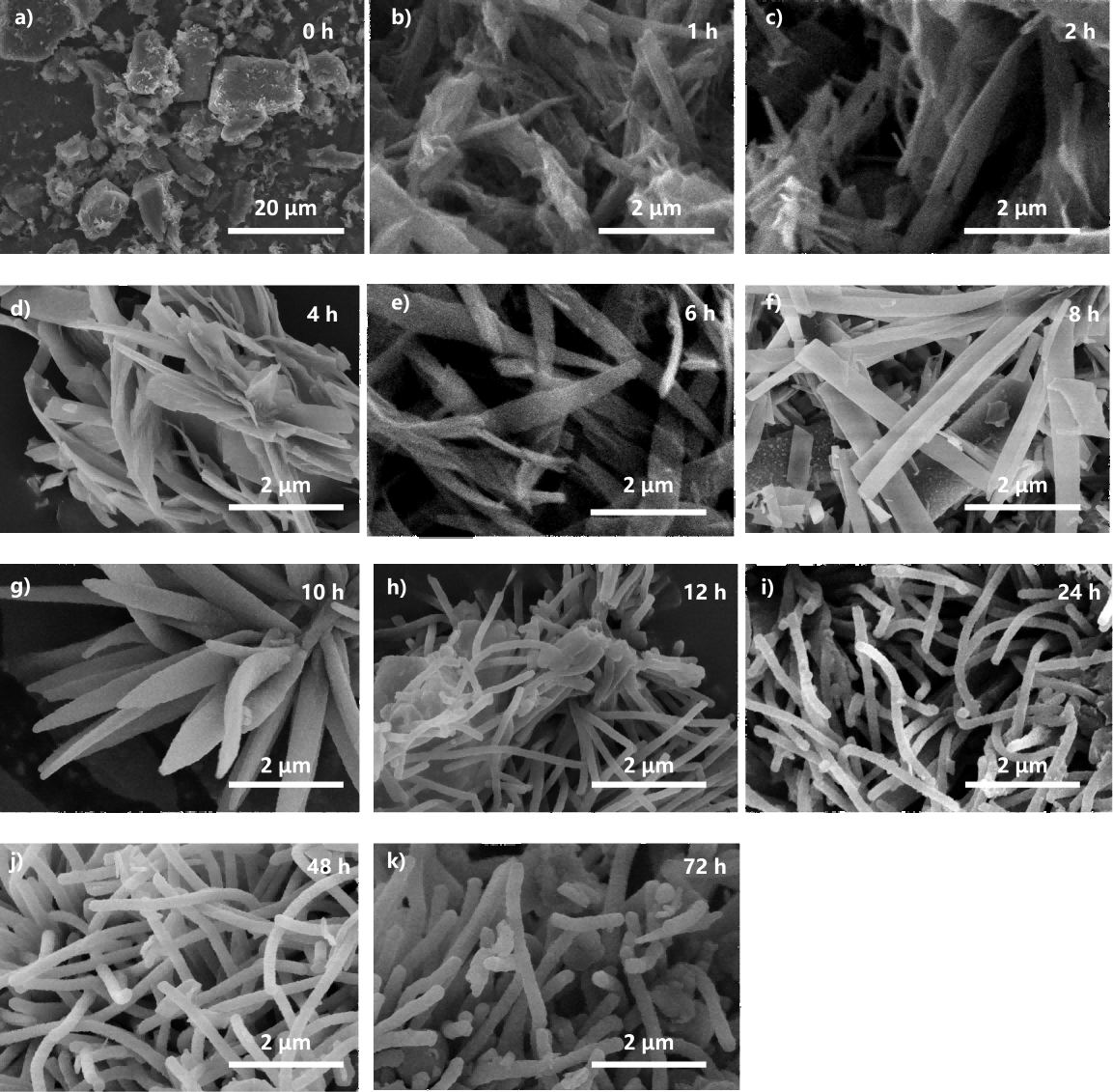


**Fig. S4** SEM images of the CNF synthesized at different time points


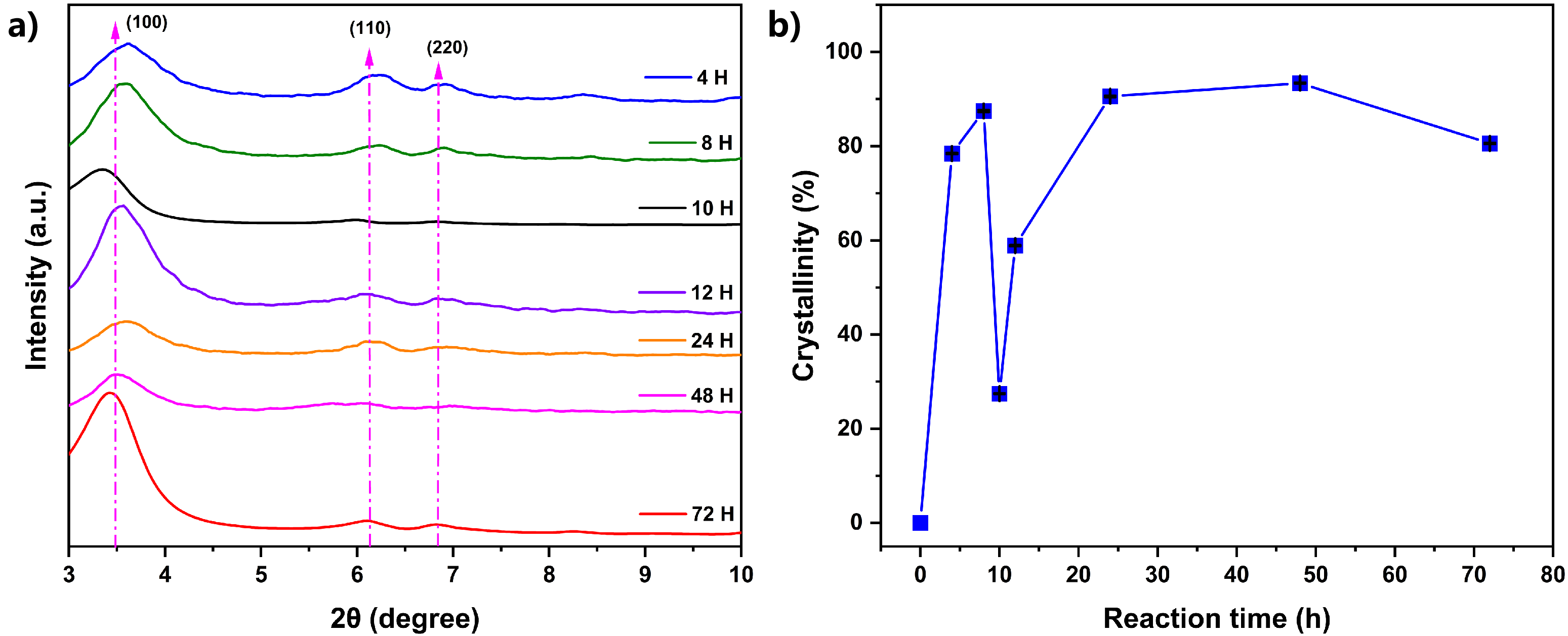


**Fig. S5 a** XRD spectra, and **b** calculated crystallinity of the CNF synthesized at different time points


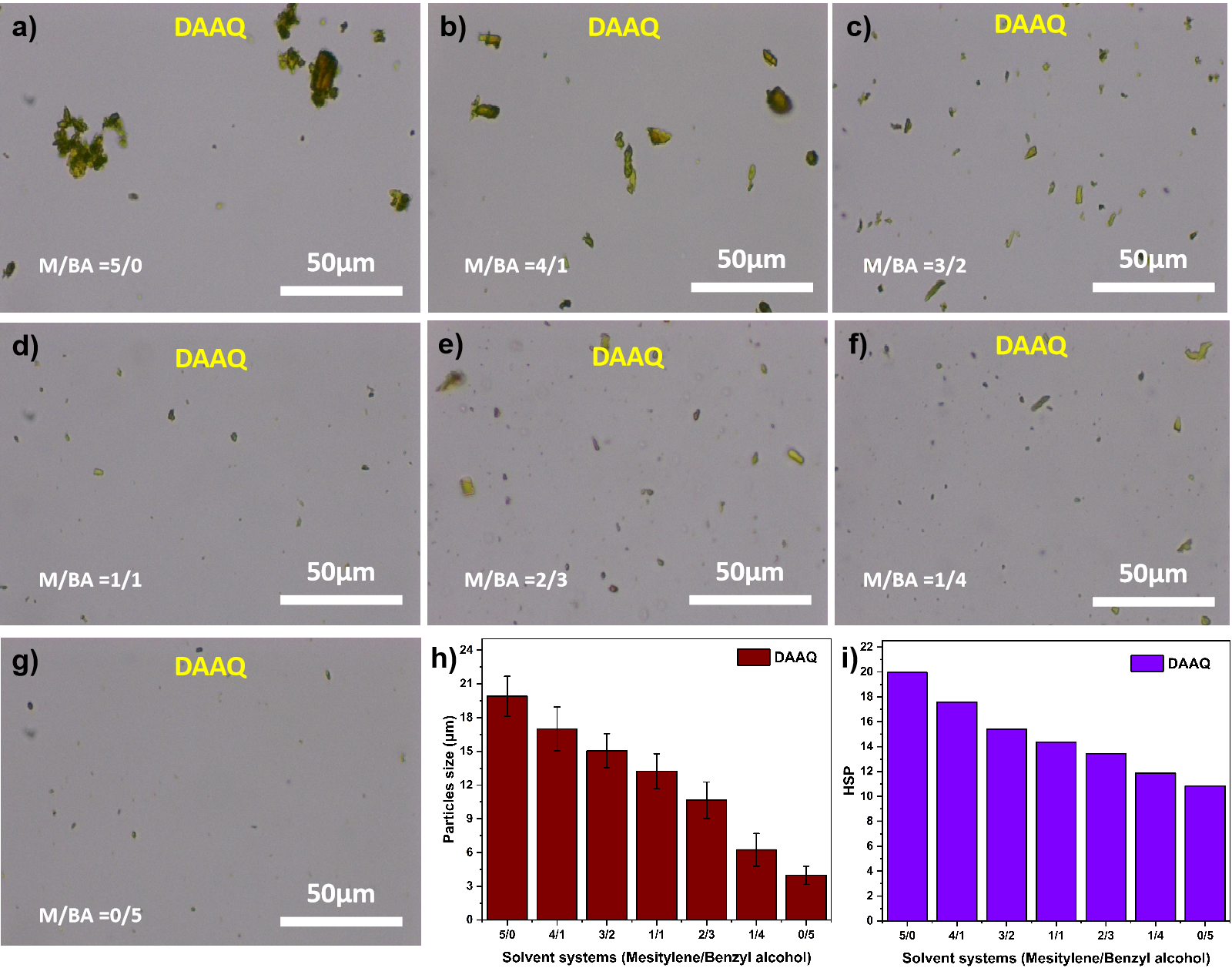
**Fig. S6** **a-g** Microscope photos of DAAQ in different M/BA systems under ultrasonication for 30 min and stirring 60 min. **h** The DAAQ particle sizes and **i** HSP data of DAAQ in different M/BA systems


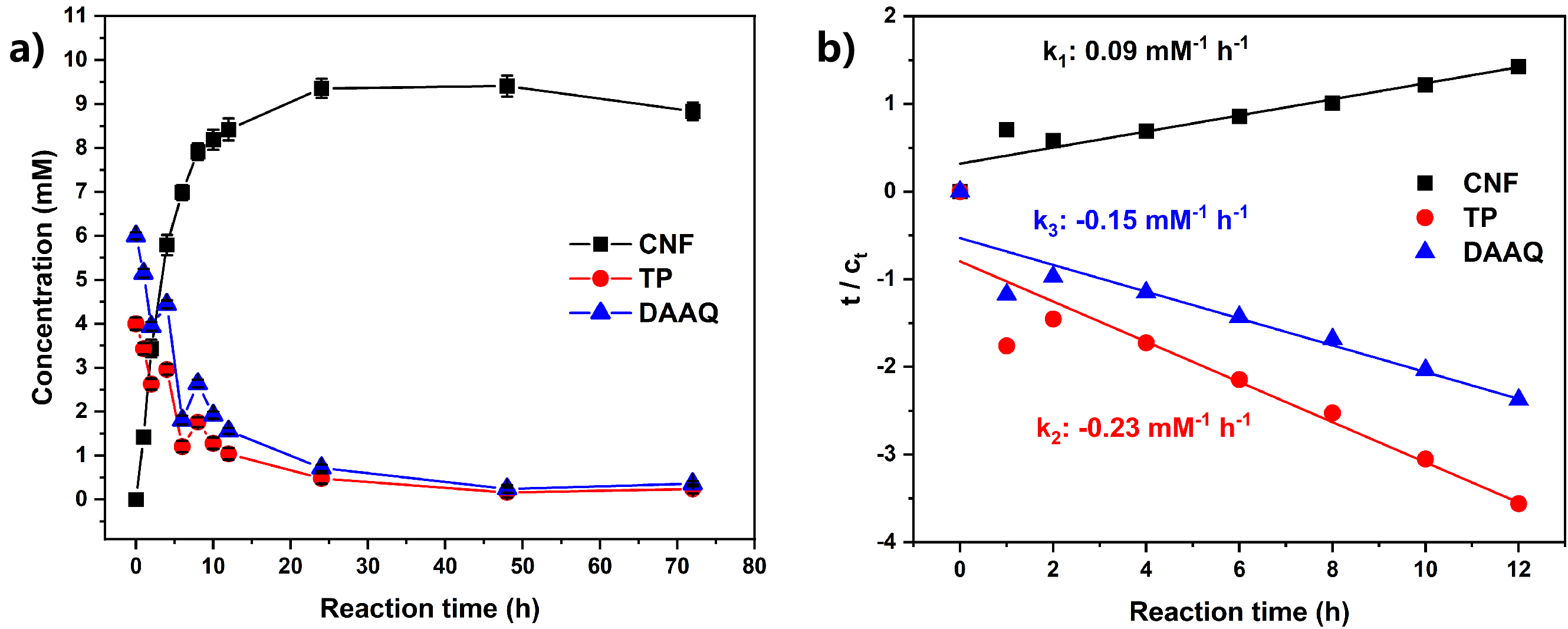


**Fig. S7 a** Correlation between the concentration changes of TP, DAAQ, and CNFs in the synthesis system and reaction time. **b** Second-order kinetics of TP and DAAQ consumption and CNF formation


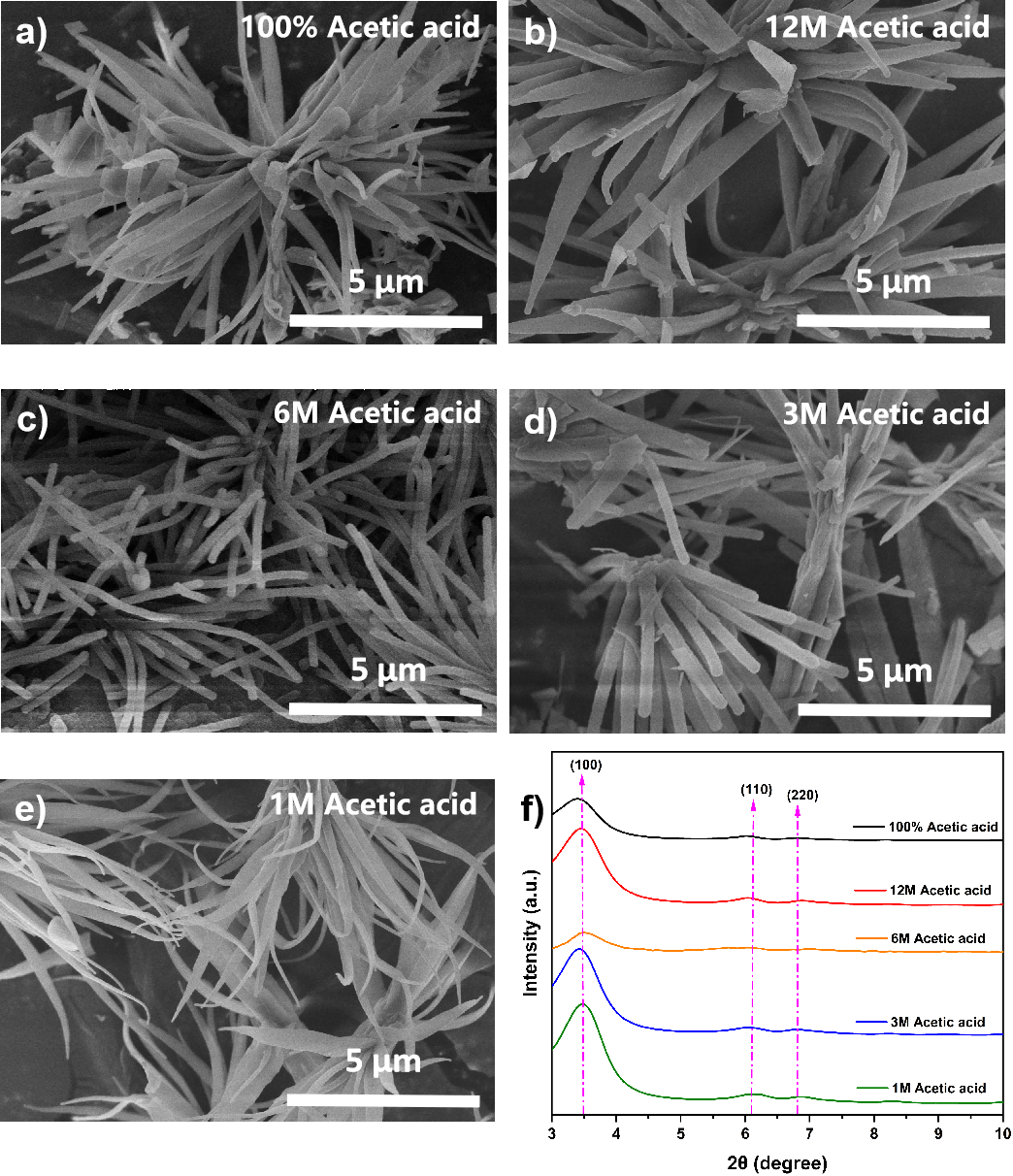


**Fig. S8** **a-e** SEM images and **f** XRD spectra of the CNF synthesized with different concentrations of catalyst


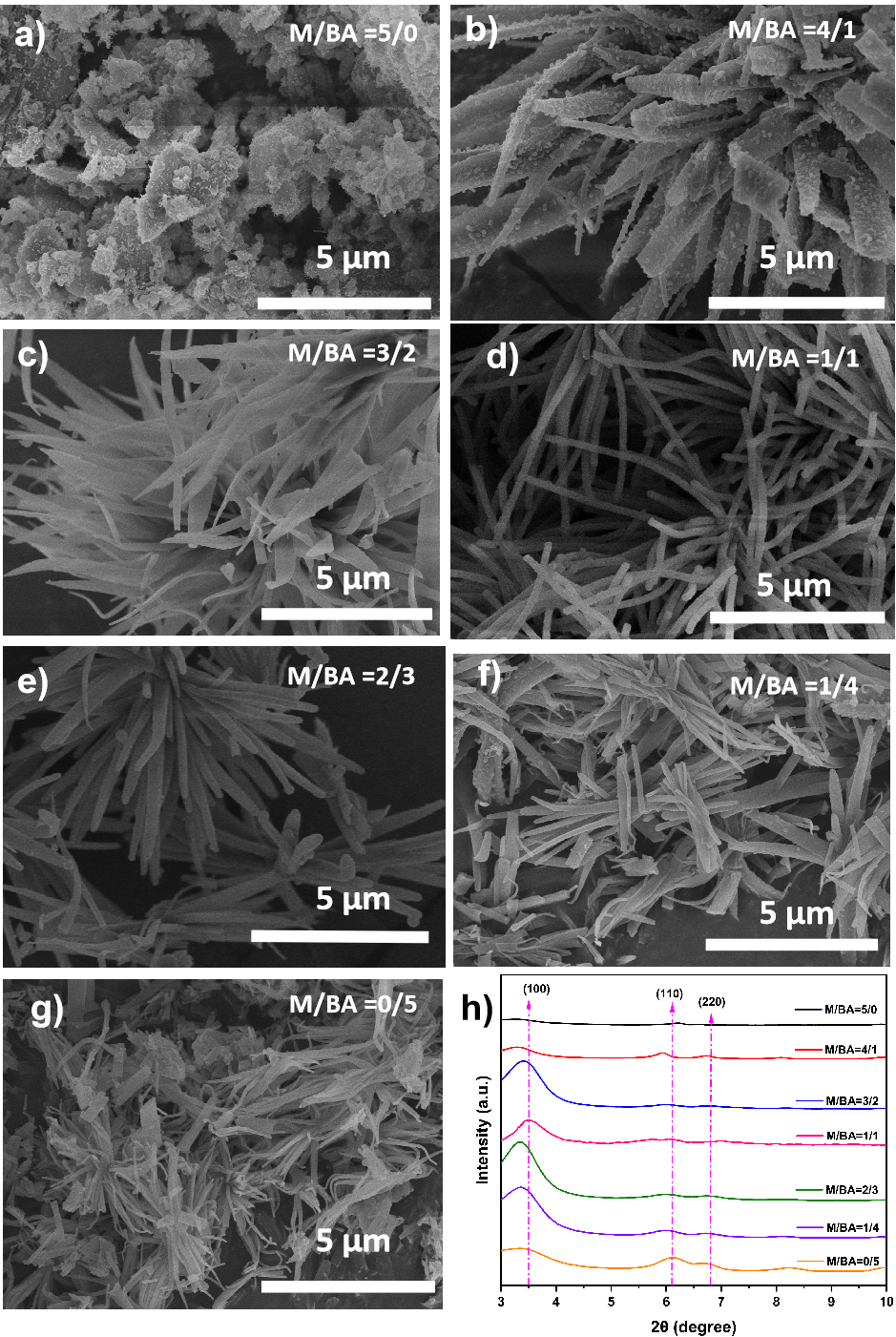


**Fig. S9** **a-g** SEM images and **h** XRD spectra of the CNF synthesized in different solvent systems


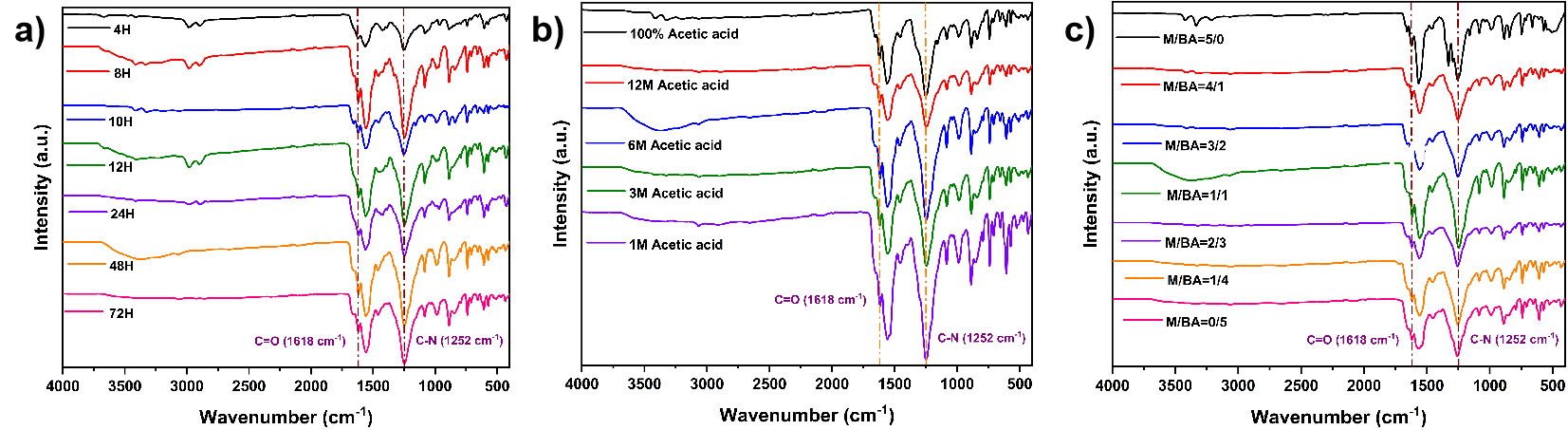


**Fig. S10** The ATR spectra of CNFs synthesized **a** at different time points, **b** with different concentrations of catalysts, and **c** in different solvent systems


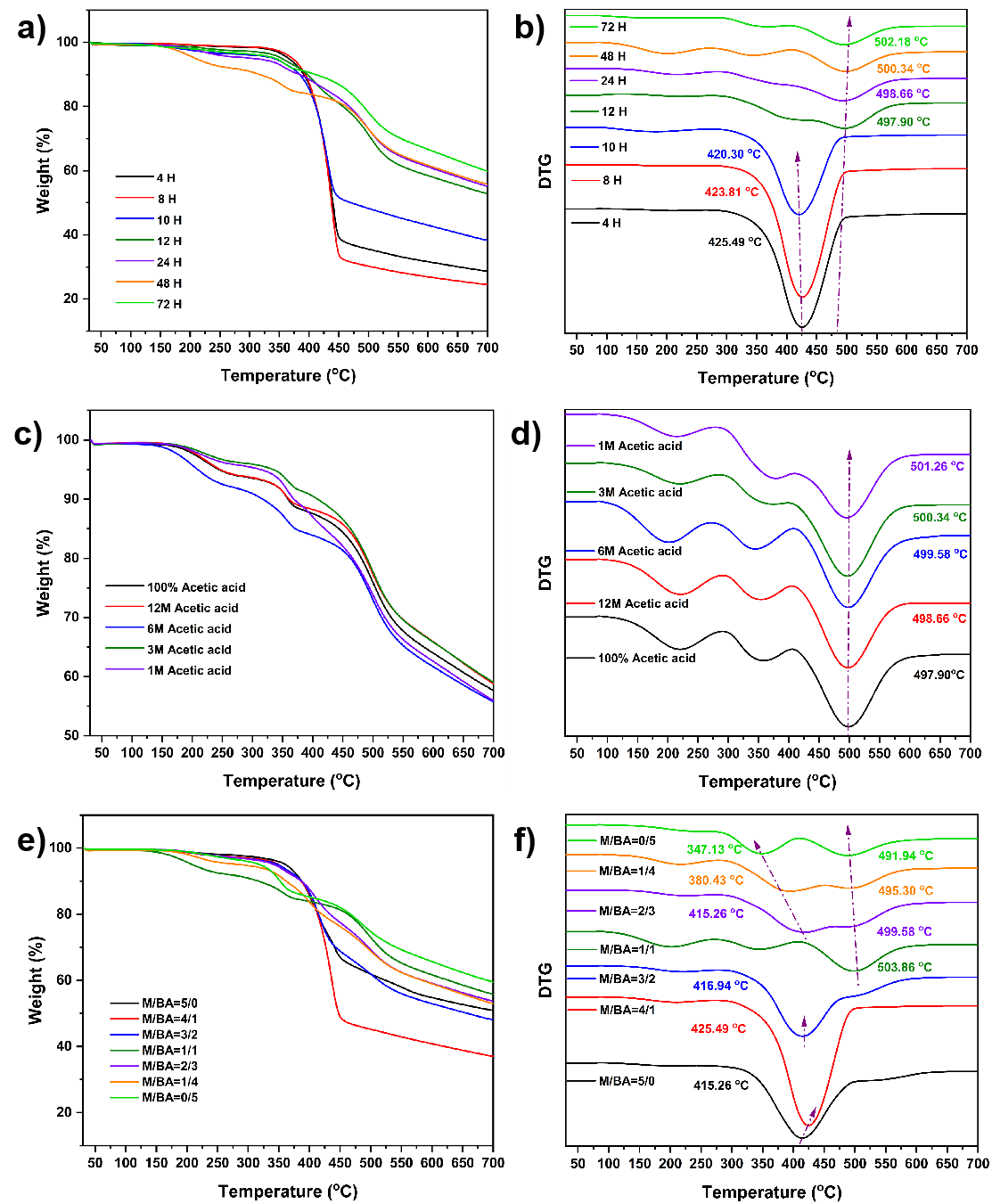


**Fig. S11** The TGA and DTG curves of CNFs synthesized **a, b** at different time points, **c, d** with different concentrations of catalysts, and **e, f** in different solvent systems


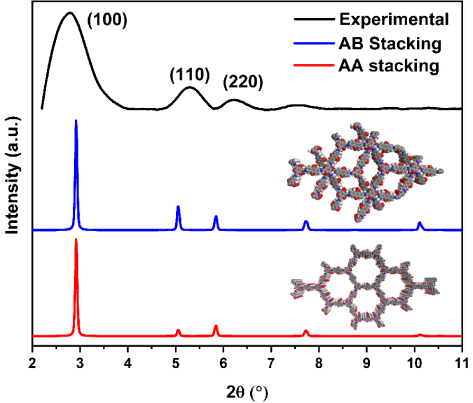


**Fig. S12** Experimental and simulated XRD spectra of the CNF


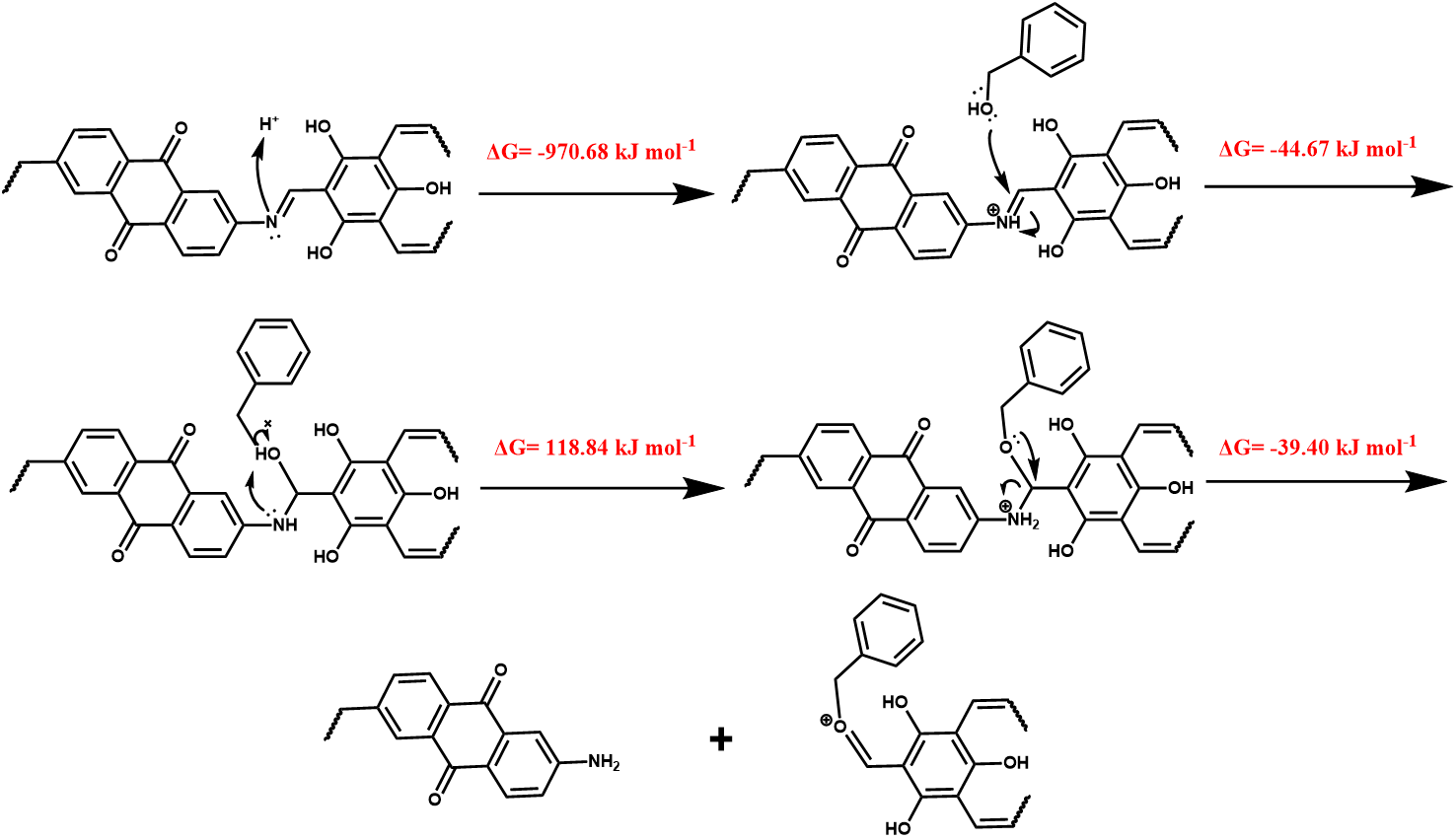


**Fig. S13** Reversed Schiff based reaction triggered by BA


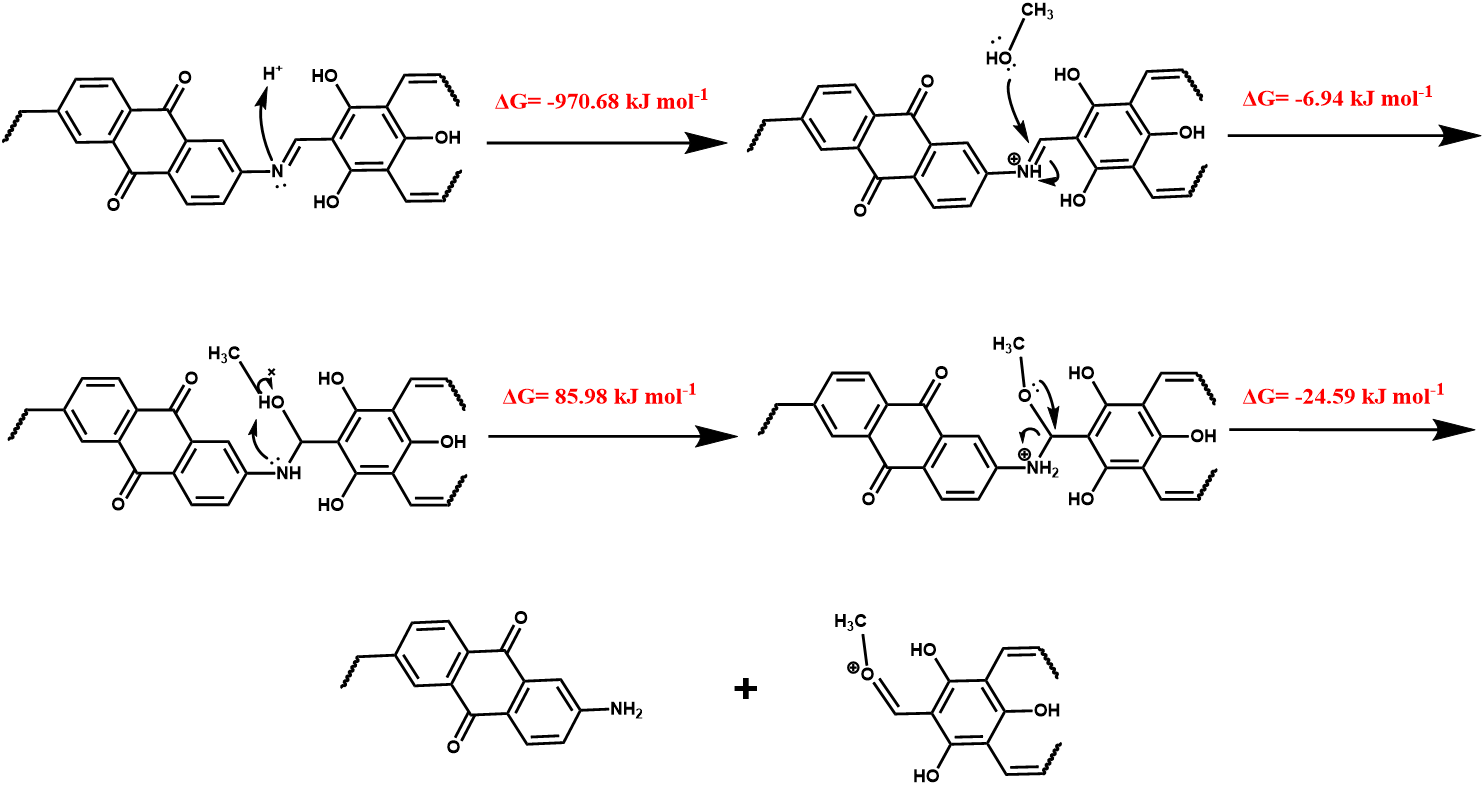


**Fig. S14** Reversed Schiff based reaction triggered by MA


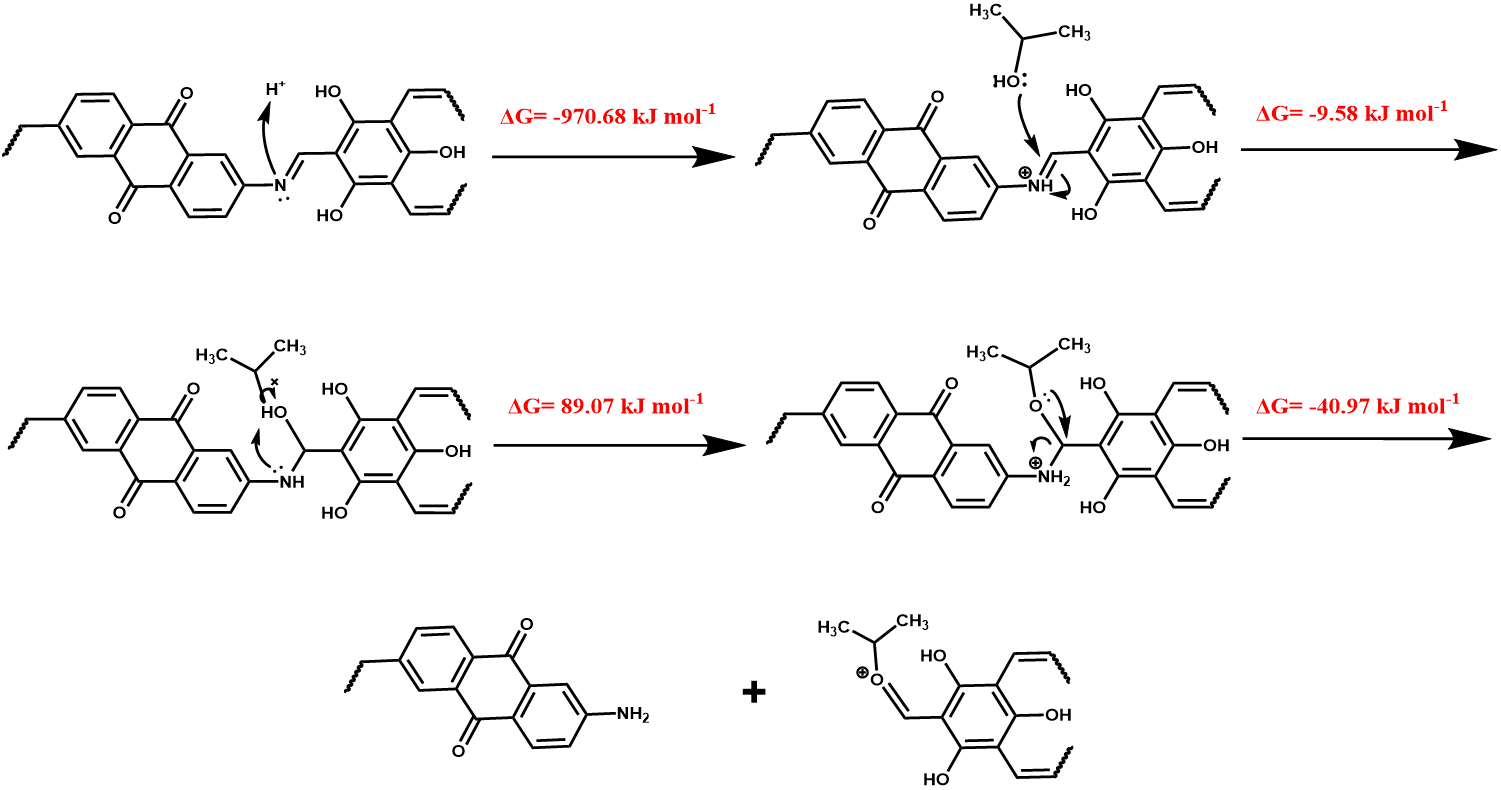


**Fig. S15** Reversed Schiff based reaction triggered by IPA


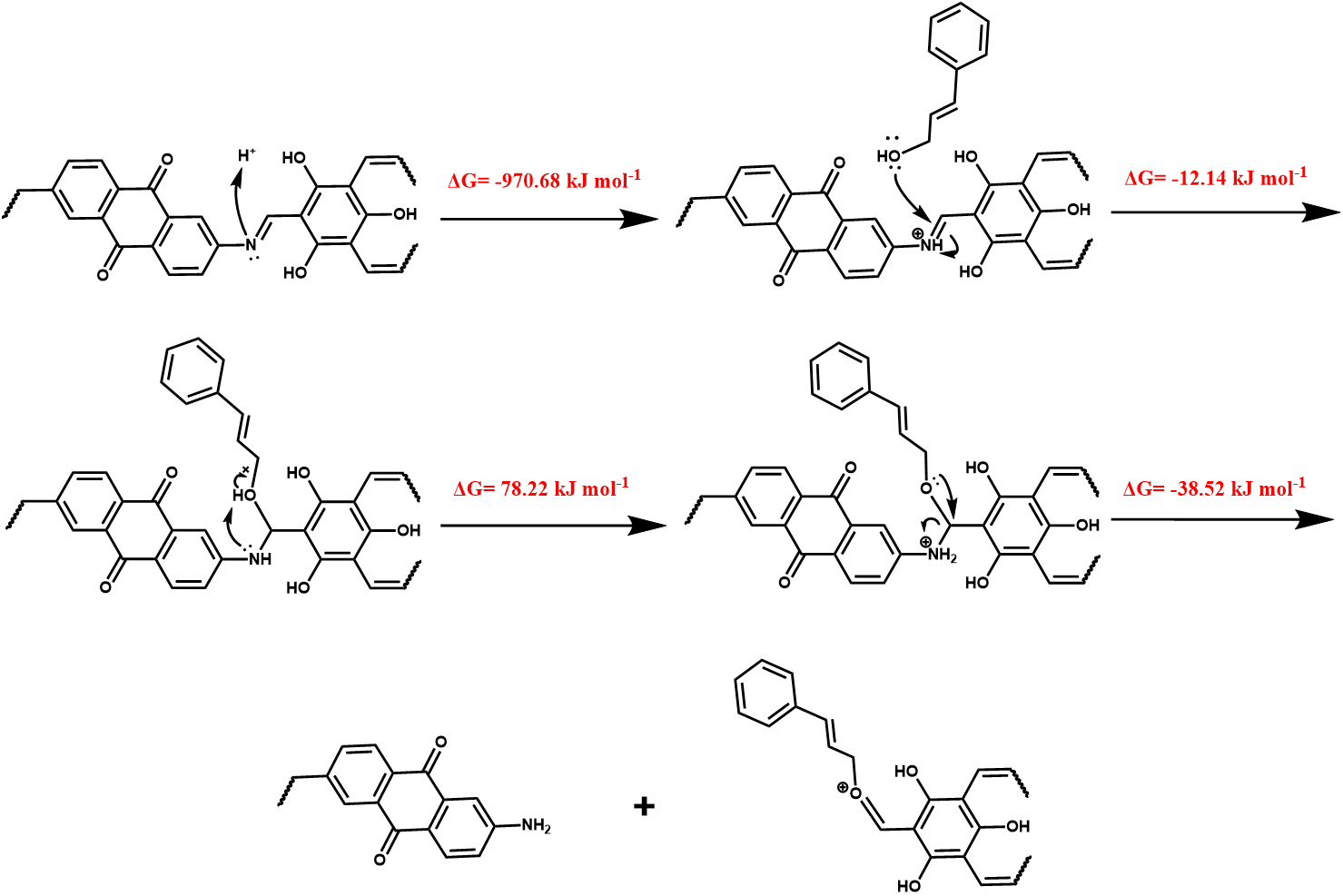


**Fig. S16** Reversed Schiff based reaction triggered by CA


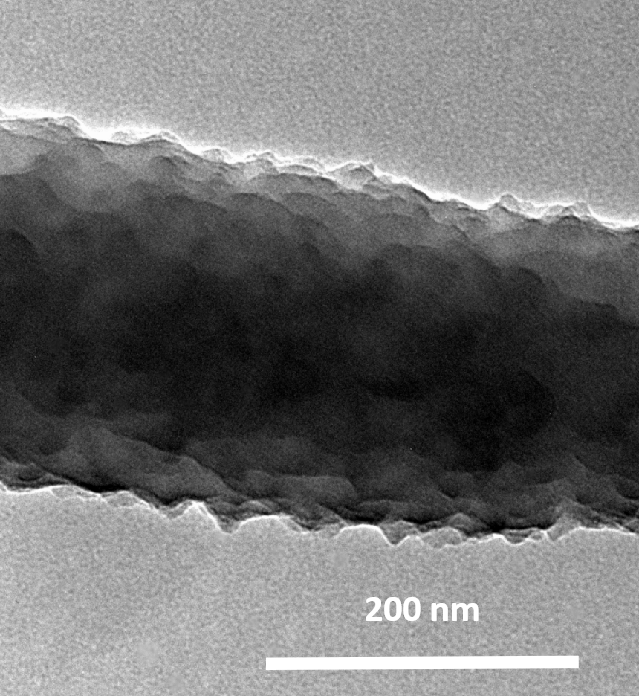


**Fig. S17** TEM image of CNF with misaligned overlapping COF grains


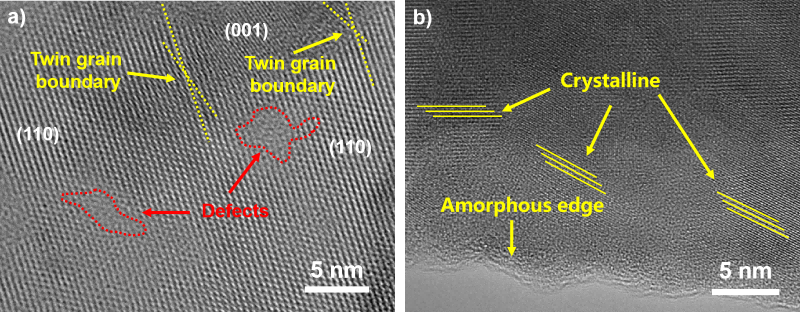


**Fig. S18** HR-TEM images CNF containing grains with different orientations and crystalline/amorphous distinct regions


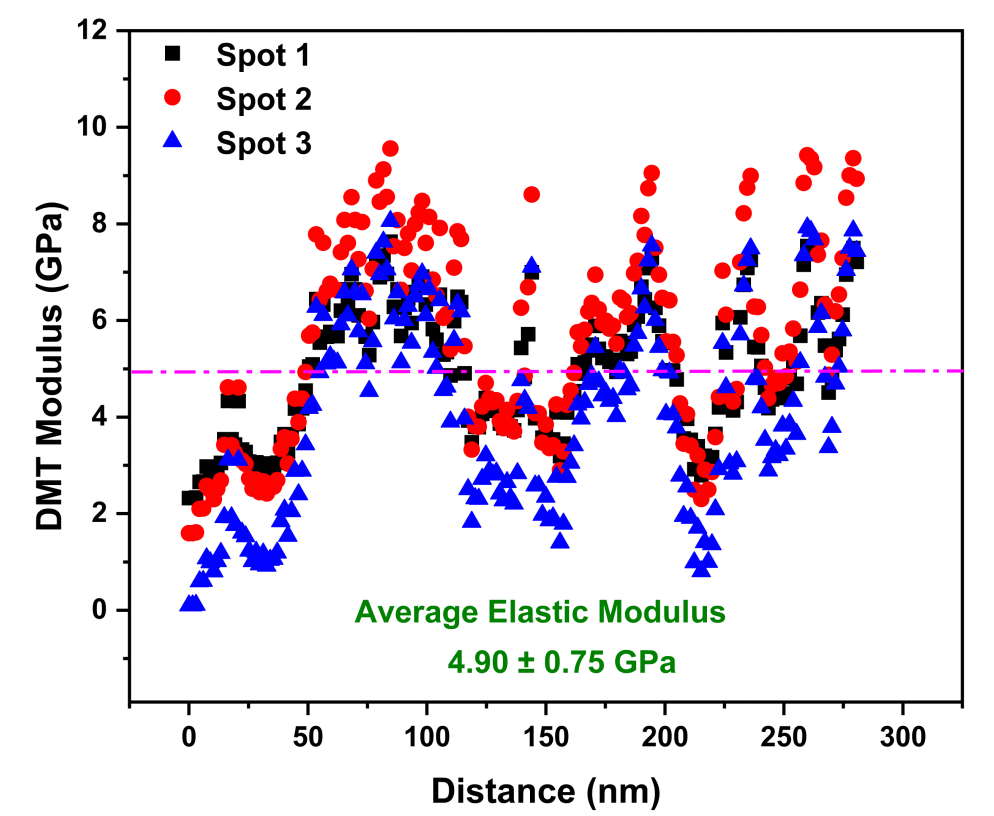


**Fig. S19** The distribution of elastic modulus along three different spots in an individual CNF


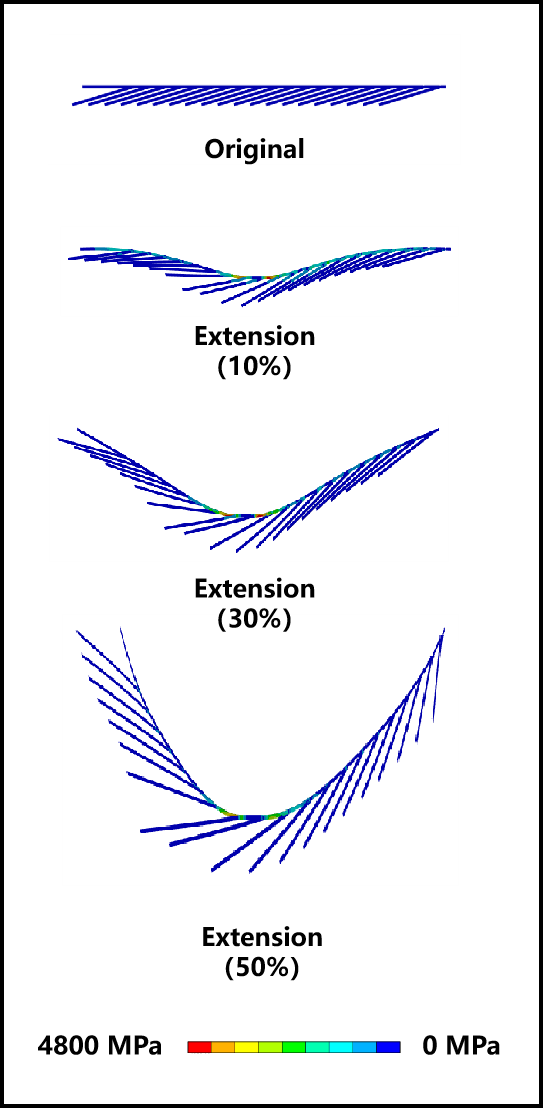


**Fig. S20** FEA simulation of the extension side of the CNF under dynamic bending


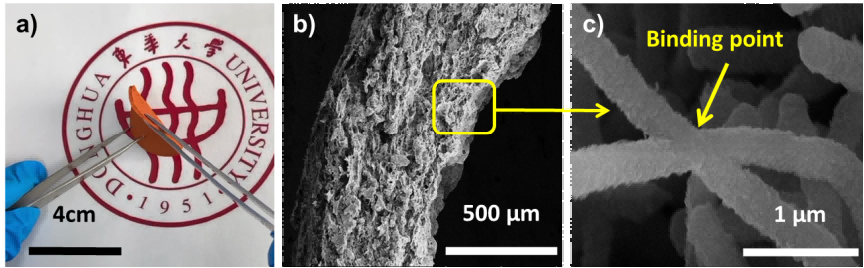


**Fig. S21** **a** The optical image of CNF-M with good flexibility. **B-c** SEM images of the cross-section of CNF-M and the binding points between CNFs caused by photoinduced crosslinking


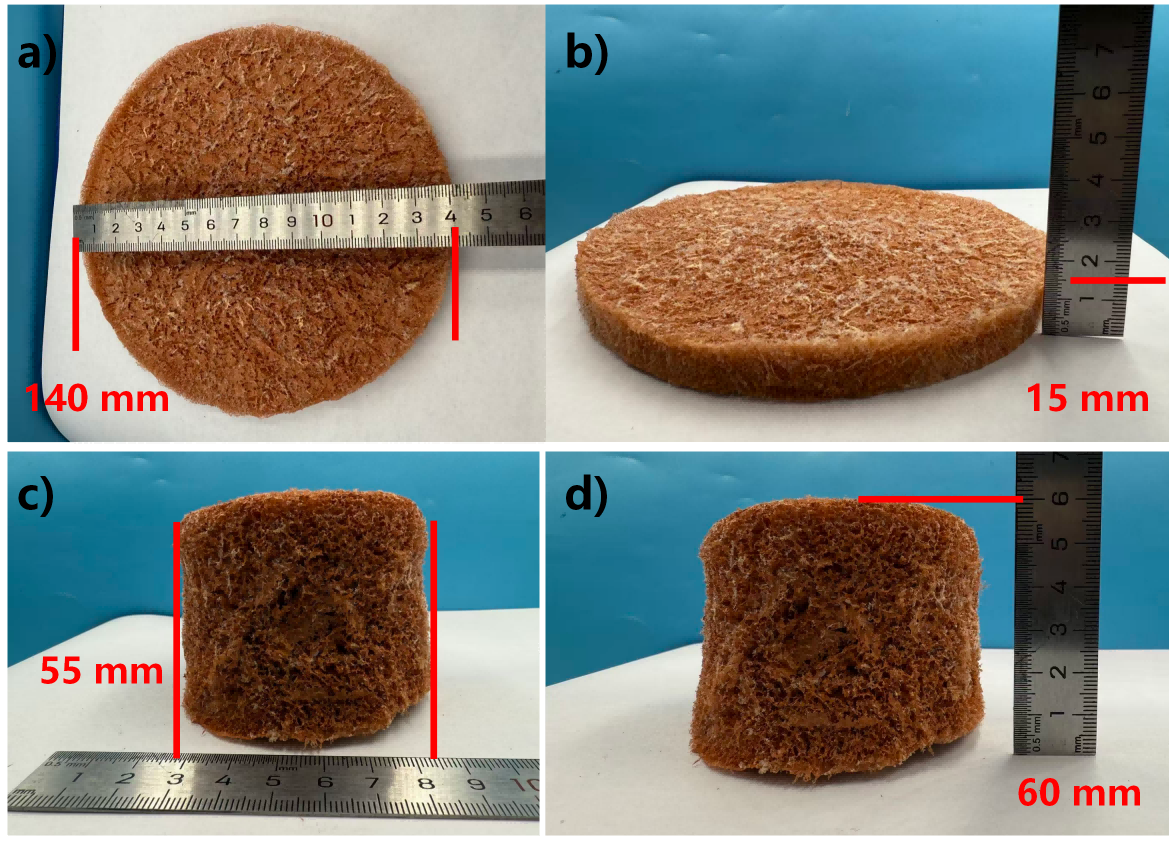


**Fig. S22** Photographs of the large-size CNF-As in size of **a, b** 140 mm×15 mm (D×H), and **c, d** 55 mm×60 mm (D×H)


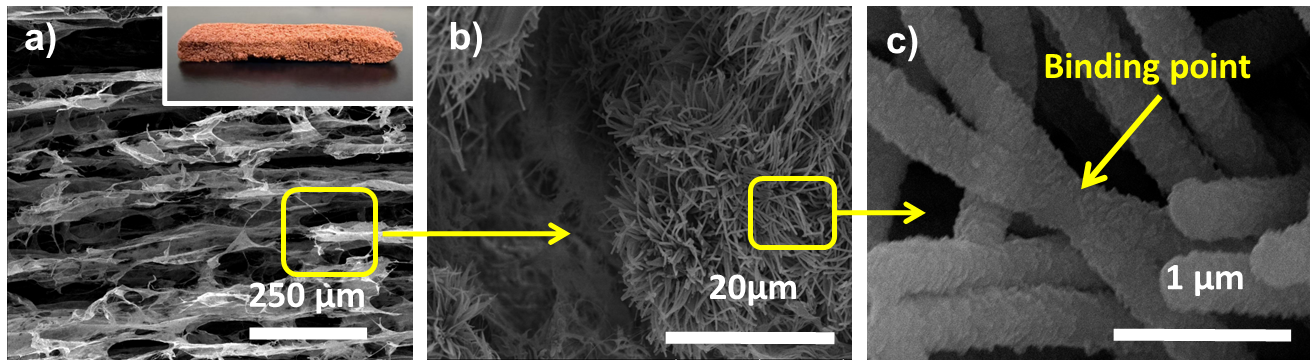


**Fig. S23** The photograph and SEM image of the cross-section of the laminated CNF-A under different magnifications. The binding point between CNFs is attributed to the photoinduced chemical crosslinking


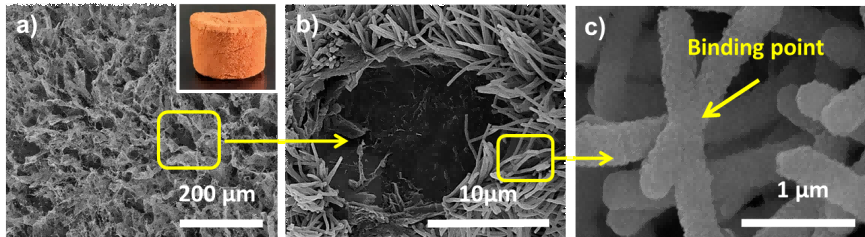


**Fig. S24** The photograph and SEM images of the cross-section of the isotropic CNF-A under different magnifications. The binding point between CNFs is attributed to the photoinduced chemical crosslinking


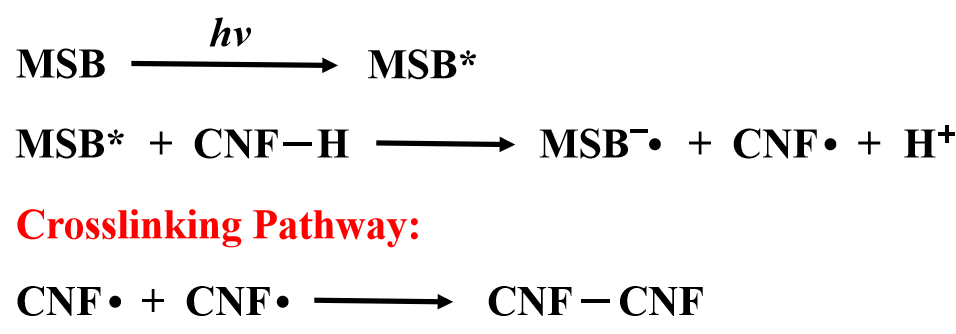


**Fig. S25** The photoinduced chemical crosslinking reaction of CNFs [S1]

**
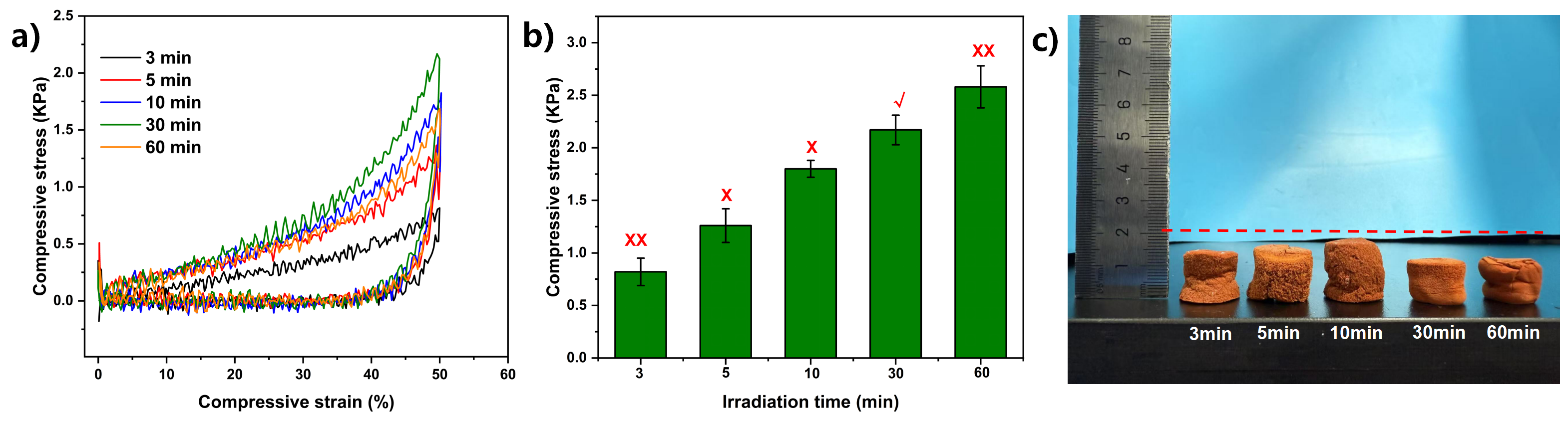
**

**Fig. S26** **a** The stress-strain curves (ε=50%) **b** maximum stress at ε=50%, and **c** the photographs of CNF-As after compression in a dry condition with different irradiation times during photo-crosslinking. The original height of the CNF-As were 20 mm. √ indicates that CNF-A maintains structural integrity after compression. X denotes the formation of minor cracks or presence of slight deformation after compression. XX refers to the formation of large cracks or presence of sever deformation after compression.


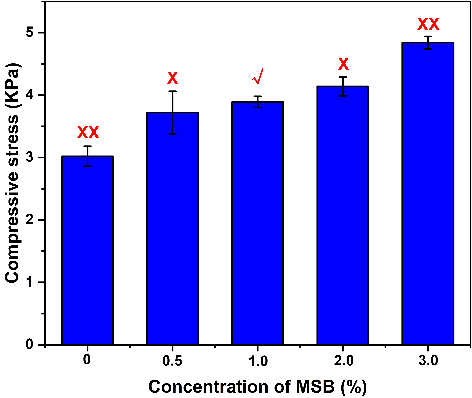


**Fig. S27** Compression stress of CNF-As with different MSB concentrations. The irradiation time was set as 10 min. √ indicates that CNF-A maintains structural integrity after compression. X denotes the formation of minor cracks or presence of slight deformation after compression. XX refers to the formation of large cracks or presence of sever deformation after compression.


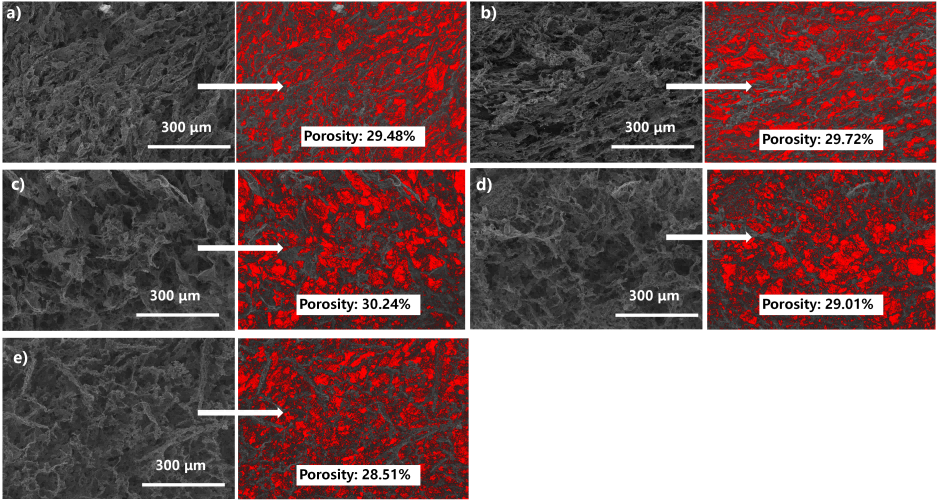


**Fig. S28** SEM and ImageJ converted images of CNF-As with different MSB concentrations: **a** 0%, **b** 0.5%, **c** 1.0%, **d** 2.0%, and **e** 3.0%. Irradiation time=10 min. No big difference in porosity of CNF-As was noticed when varying the MSB concentration from 0~3.0%. Considering the crosslinking efficiency and economy, 1.0% of MSB was set as the optimal condition


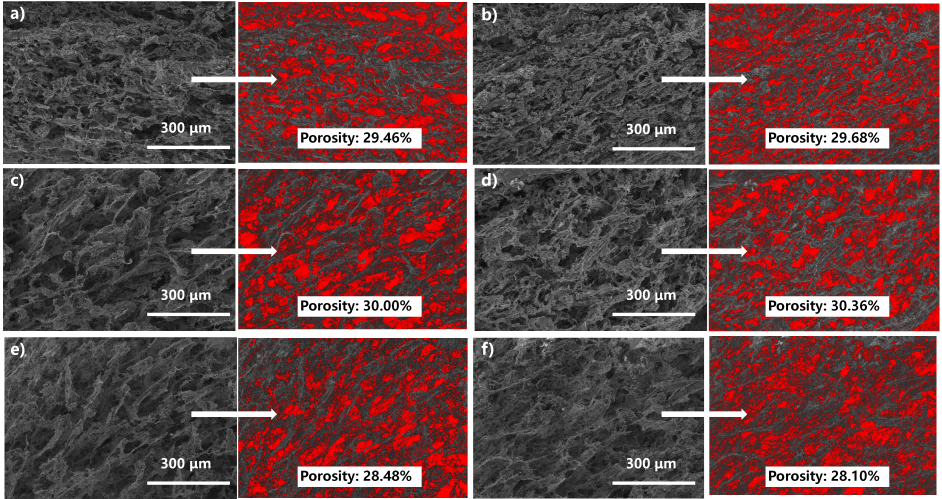


**Fig. S29** SEM and ImageJ converted images of CNF-As with different irradiation times during photo-crosslinking: **a** 0 min, **b** 3 min, **c** 5 min, **d** 10 min, **e** 30 min, and **f** 60 min. MSB concentration=1.0%. Slightly decrease in porosity of CNF-As by ~2% was noticed when varying the irradiation time from 0~60 min. Irradiation time of 10 min achieved the optimal relationship between mechanical properties and porosity.


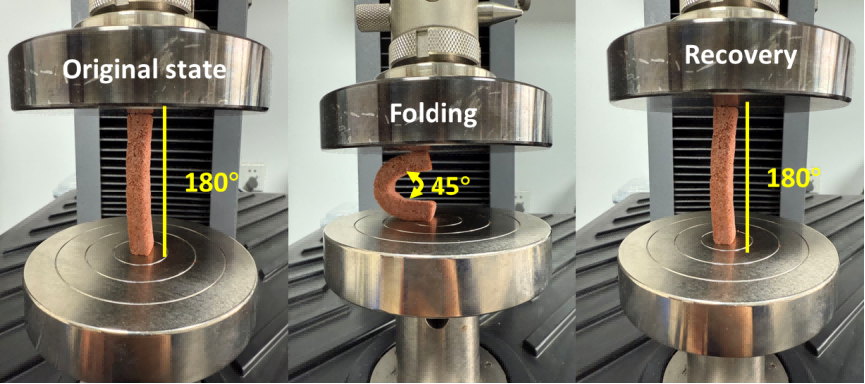


**Fig. S30** The photographs of CNF-A, which can be folded and recovered without forming any cracks


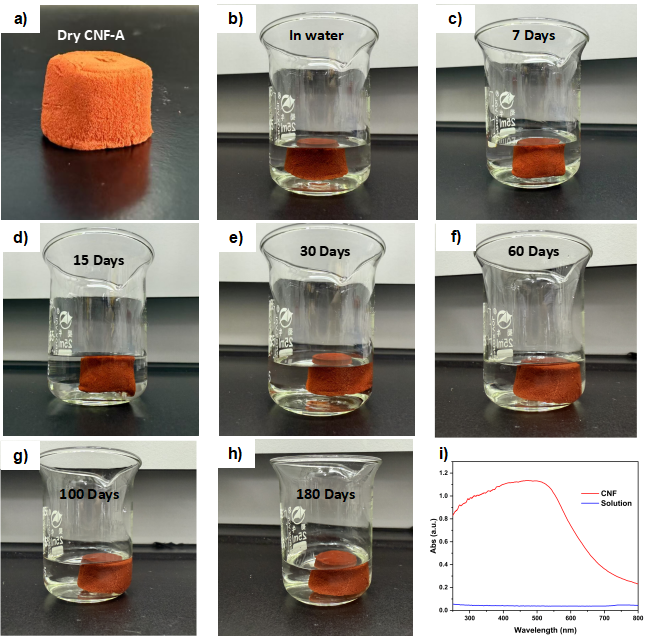


**Fig. S31** Photographs of CNF-A **a** in a dry phase and **b-h** structural stability in water with different immersion durations. **I** UV-vis spectra of CNF-A suspension, and the solution after immersion for 180 days. The CNF suspension presented a broad adsorption at 250-600 nm, while no such adsorption was found in the solution after CNF-A immersion for 180 days.


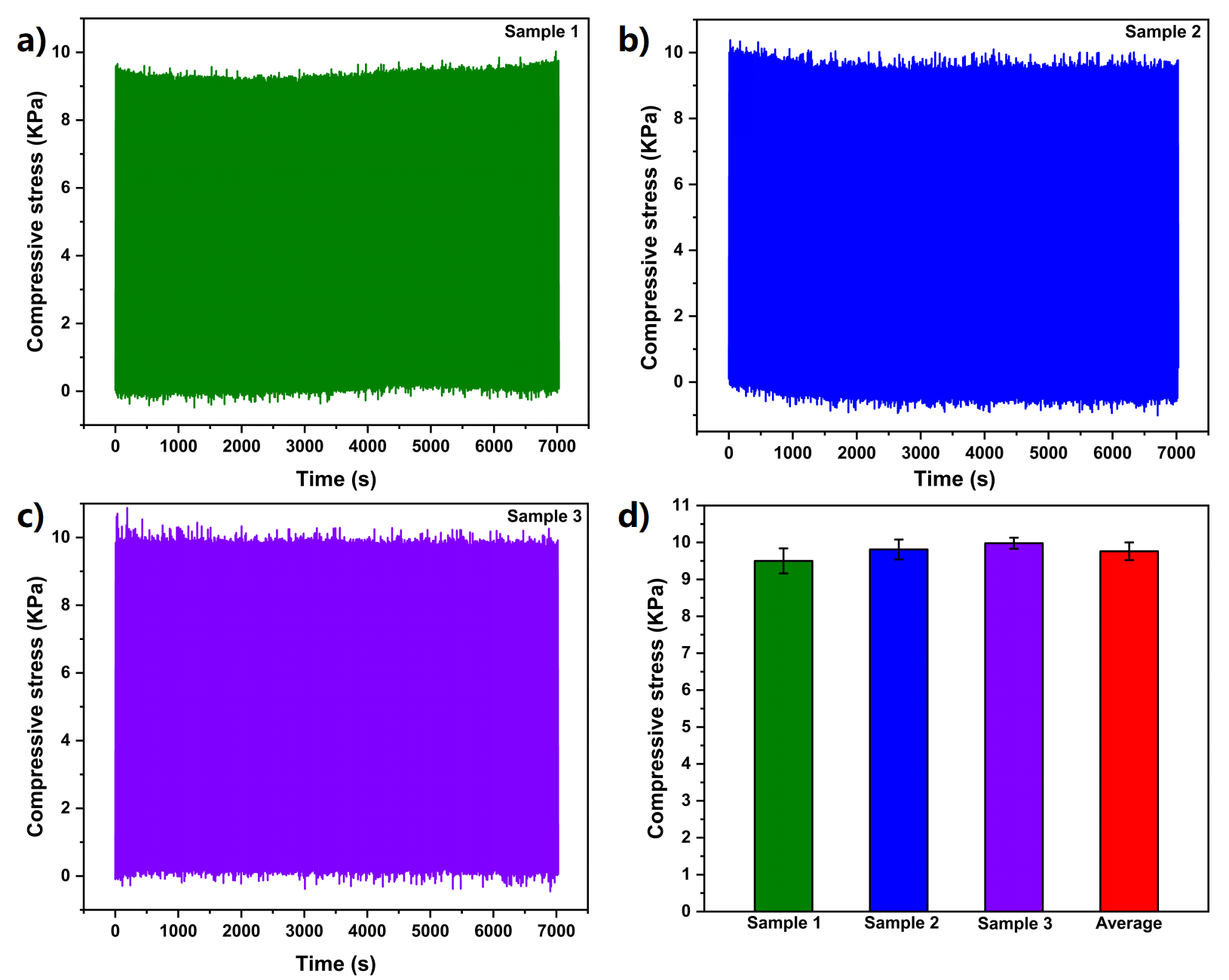


**Fig. S32** **a-c** Stress-time compression curves of different CNF-A samples for 500 cycles in water. **D** Averaged compressive stresses of CNF-As


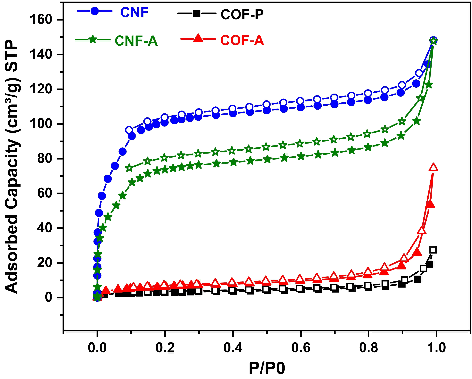


**Fig. S33** The N_2_ sorption isotherms of different materials at 77 K. CNF and CNF-A both exhibited a type I/II character with sharp adsorption at lower (P/P_0_ < 0.1) and higher (P/P_0_ > 0.9) partial pressures, indicating the presence of micropores and mesopores in their structures. The presence of hysteresis loop also described specific adsorption behavior of a mesoporous material.


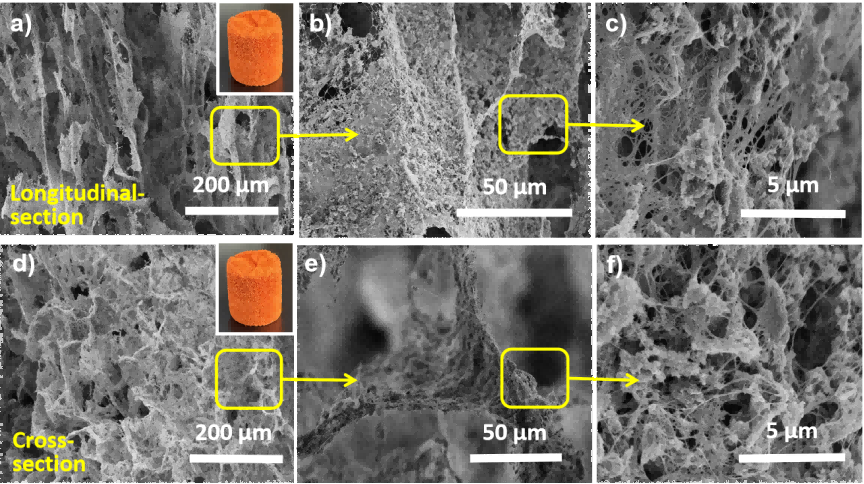


**Fig. S34** The SEM images of **a-c** longitudinal-section and **d-f** cross-section of COF-A. The inserted photographs display the optical image of COF-A


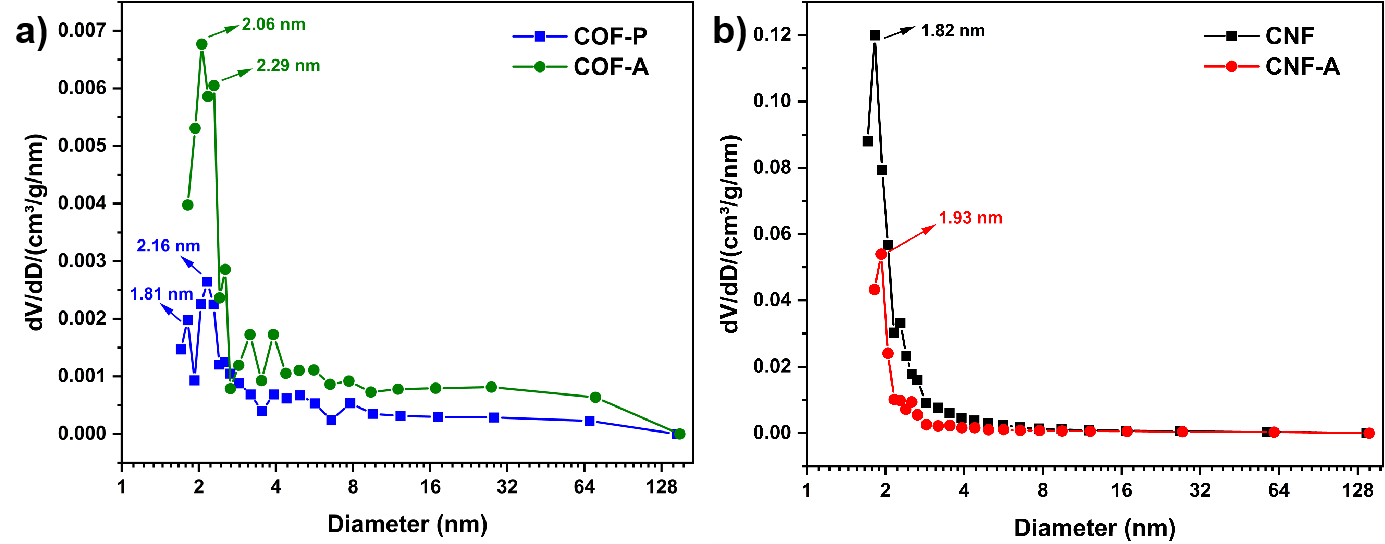


**Fig. S35** The pore size distribution of different materials


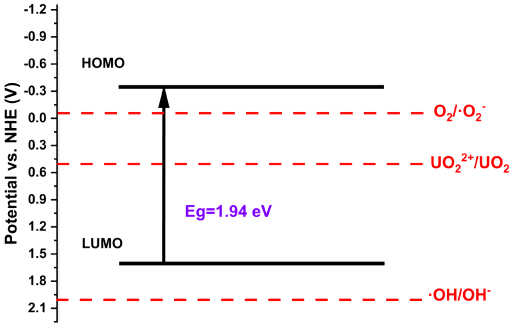


**Fig. S36** Schematic illustration of the mechanism of photocatalytic reduction of UO_2_^2+^ to UO_2_ by CNF-A

The photoexcitation of the benzophenone moiety in the CNFs is essential to the reduction of U(VI) to U(IV). The benzophenone moiety exhibits strong absorption in the ultraviolet (UV) to visible light range due to its conjugated π-system and carbonyl group. According to reported literature [S2, S3, S39], electrons occupying the HOMO orbital, which is the n electrons in the carbonyl group of benzophenone, are promoted into the antibonding π* orbitals (LUMO) in the conjugated benzophenone ring. When excited-state electrons return from the LUMO orbital to the HOMO (i.e., the excited state of CNF returns to its ground state), they can be captured by surrounding U(VI) ions because of the energy matching between the HOMO/LUMO levels of CNFs and the redox potential of UO_2_^2+^/UO_2_. This process realizes the reduction reaction of U(VI) to U(IV) by the benzophenone group in CNFs.


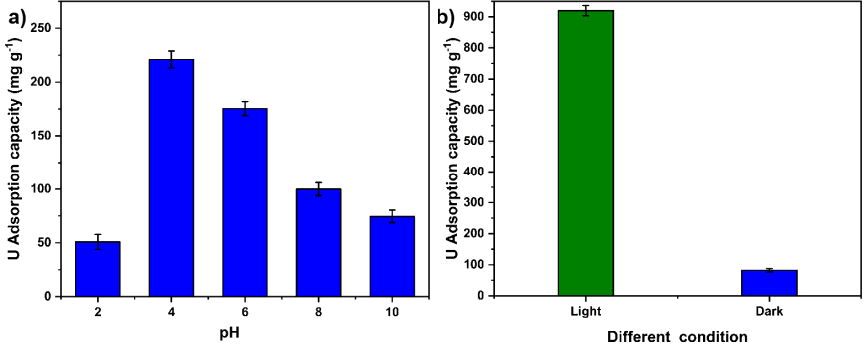


**Fig. S37** **a** The adsorption amount of CNF-A from U-spiked distilled water (initial concentration=32 ppm) with different Ph. **B** Adsorption amount of U from U-spiked distilled water under visible light irradiation and dark incubation (initial concentration=128 ppm)


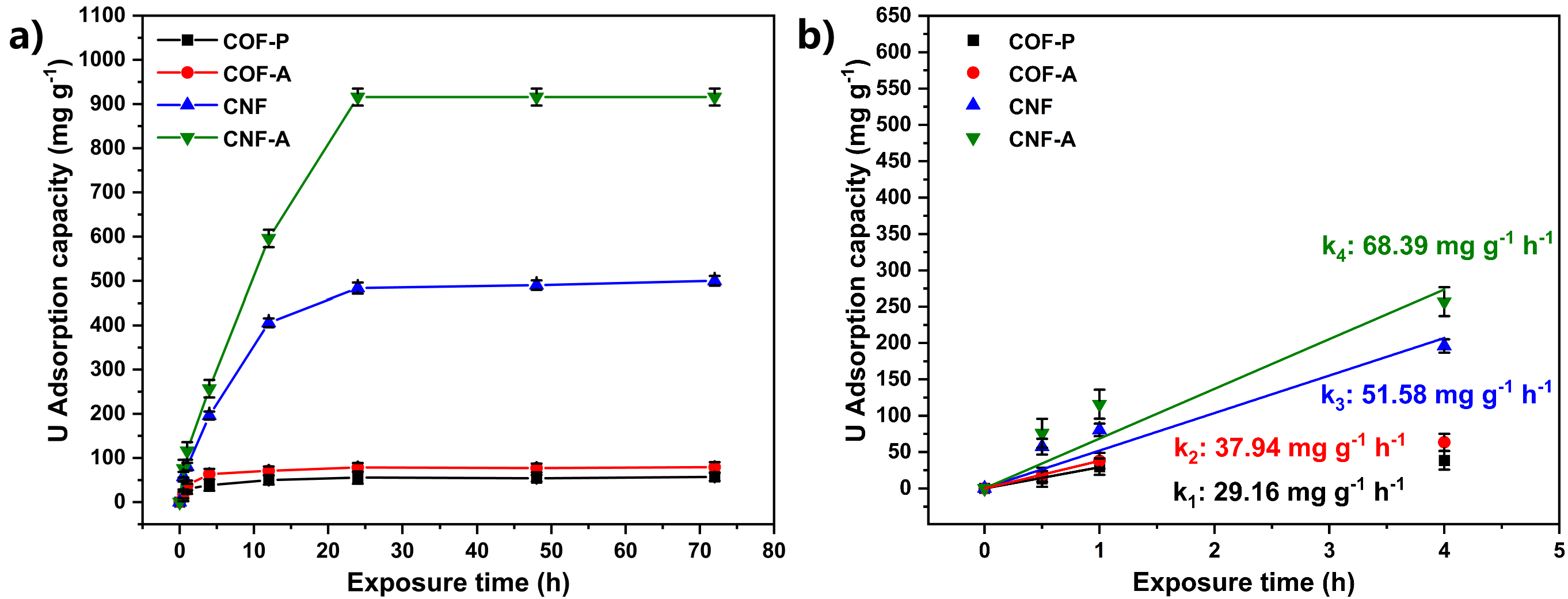


**Fig. S38 a** U absorption amounts versus time of different materials. **b** Initial adsorption rate of different materials


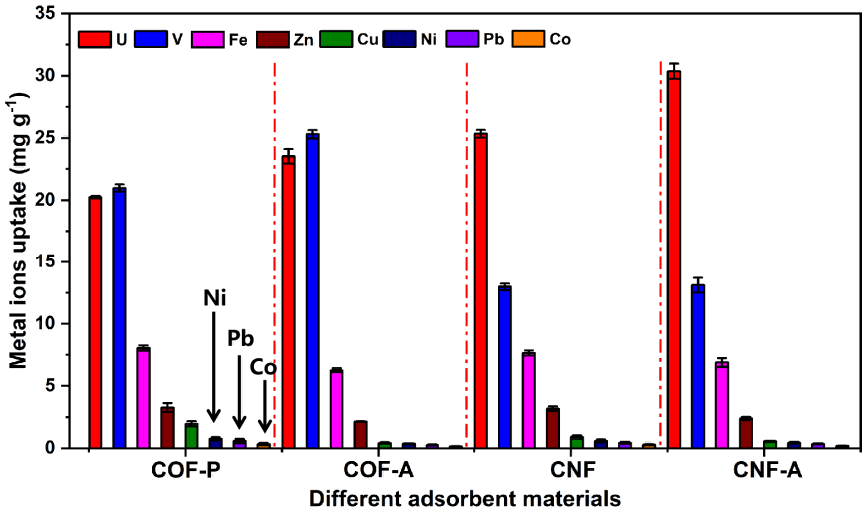


**Fig. S39** Adsorption amount of competitive metal ions in distilled water by different materials under visible light irradiation for 24 h


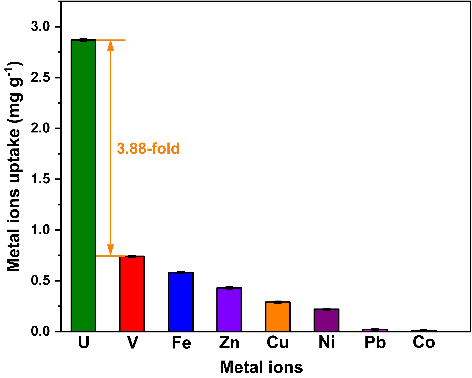


**Fig. S40** Adsorption amount of U and competitive metal ions by CNF-A in natural seawater after 15 days


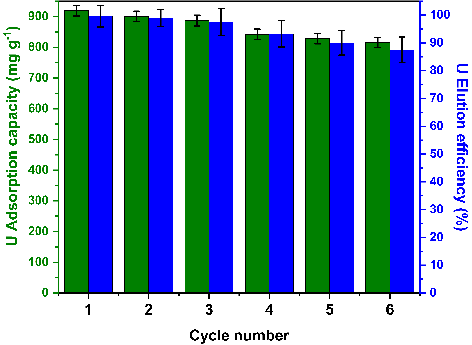


**Fig. S41** U adsorption amount (initial concentration=128 ppm) and elution efficiency in six successive adsorption-desorption cycles under visible light irradiation by CNF-As

**S3 Supplementary Tables**

**Table S1** Size characteristics of previously reported fiber-shaped COFs

| **Labels** | **Materials** | **Length (nm)** | **L/D ratio** | **Refs.** |
| --- | --- | --- | --- | --- |
| 1 | CPPs | 515 | 5.72 | [S4] |
| 2 | TPT-DAHQ COF | 7400 | 7.40 | [S5] |
| 3 | TfBD | 5000 | 62.50 | [S6] |
| 4 | MF-COF-1 | 2500 | 35.71 | [S7] |
| 5 | TAPB-BTCA 12C | 20000 | 66.70 | [S8] |
| 6 | N_3_-COF_100_ | 2000 | 40.00 | [S9] |
| 7 | COF-300 | 10000 | 2.00 | [S10] |
| 8 | COF-300 | 20000 | 1.54 | [S10] |
| 9 | COF-300 | 60000 | 3.00 | [S10] |
| 10 | LZU-79 | 100000 | 12.50 | [S10] |
| 11 | LZU-111 | 50000 | 1.00 | [S10] |
| / | **CNF** | **22670** | **103.05** | **This work** |

**Table S2** The HSP values and the product yields of CNF obtained from different solvent systems

| **Solvent system (v/v)** | **δD** | **δP** | **δH** | **HSP distance to DAAQ** | **HSP distance to TP** | **HSP distance to COF** | **Yield** |
| --- | --- | --- | --- | --- | --- | --- | --- |
| M/BA 5/0 | 18.00 | 0.60 | 0.60 | 19.95 | 26.01 | 22.91 | 48.60% |
| M/BA 4/1 | 18.08 | 1.74 | 3.22 | 17.58 | 23.43 | 20.41 | 72.11% |
| M/BA 3/2 | 18.16 | 2.88 | 5.84 | 15.38 | 20.92 | 18.02 | 85.92% |
| M/BA 1/1 | 18.20 | 3.45 | 7.15 | 14.37 | 19.70 | 16.88 | 94.11% |
| M/BA 2/3 | 18.24 | 4.02 | 8.46 | 13.44 | 18.51 | 15.79 | 89.51% |
| M/BA 1/4 | 18.32 | 5.16 | 11.08 | 11.87 | 16.25 | 13.79 | 82.53% |
| M/BA 0/5 | 18.40 | 6.30 | 13.70 | 10.84 | 14.22 | 12.14 | 57.75% |
| M/MA 3/2 | 16.68 | 5.28 | 9.28 | 14.32 | 20.27 | 16.68 | 82.45% |
| M/IPA 2/3 | 16.68 | 3.90 | 10.08 | 15.09 | 20.65 | 17.24 | 86.15% |
| M/CA 2/3 | 18.66 | 3.84 | 8.04 | 13.28 | 20.80 | 16.58 | 76.27% |

**Table S3 ΔG for each step of the alcohol-triggered reverse Schiff base reaction**

| **Alcohols** | **Step 1**  **(kJ mol^-1^)** | **Step 2**  **(kJ mol^-1^)** | **Step 3**  **(kJ mol^-1^)** | **Step 4**  **(kJ mol^-1^)** |
| --- | --- | --- | --- | --- |
| BA | -940.68 | -44.67 | 118.84 | -39.40 |
| MA | -940.68 | -6.94 | 85.98 | -24.60 |
| IPA | -940.68 | -9.58 | 89.07 | -40.97 |
| CA | -940.68 | -12.14 | 78.22 | -38.52 |

**Table S4** Elastic modulus of natural and synthetic fibers

| **Fibers** | **Elastic modulus (GPa)** | **Refs.** |
| --- | --- | --- |
| **CNF** | **3.50~5.50** | **This work** |
| Cotton | 5.00~12.00 | [S11] |
| Wool | 3.20~4.50 | [S12] |
| Silk | 8.90~17.40 | [S13] |
| PET | 1.07~2.50 | [S14] |
| Nylon | 1.30~1.70 | [S15] |
| PAN nanofiber | 1.10~3.50 | [S16] |
| PTFE nanofiber | 0.17~1.42 | [S17] |
| PP fiber | 14.70~19.00 | [S18] |

**Table S5** The original data of U adsorption amount by COF-P, COF-A, CNF, and CNF-A under visible light irradiation for 24 h

| **Materials** | **U Adsorption capacity (mg g^-1^)**  **(Initial conc.=128 ppm)** | | | **Average value (mg g^-1^)** | **SE** |
| --- | --- | --- | --- | --- | --- |
| COF-P | 45.02 | 50.60 | 71.18 | 55.60 | 13.78 |
| COF-A | 78.96 | 68.05 | 88.49 | 78.50 | 10.23 |
| CNF | 496.05 | 471.17 | 483.52 | 483.58 | 12.44 |
| CNF-A | 919.09 | 930.47 | 909.96 | 920.12 | 6.55 |
|  | 919.97 | 923.05 | 915.88 |  |  |
|  | 921.54 | 926.68 | 917.04 |  |  |
|  | 912.33 | 915.47 | 929.96 |  |  |

**Table S6** Performances of reported materials for UES

| **Labels** | **Materials** | **U Adsorption**  **capacity**  **(mg g^-1^)** | **Initial**  **concentration**  **(ppm)** | **Solution system** | **Refs.** |
| --- | --- | --- | --- | --- | --- |
| a | MITpBD | 800.20 | 150 | U-spiked seawater | [S19] |
| b | UiO-66-3C4N | 654.99 | 128 | U-spiked simulated seawater | [S20] |
| c | Ti_3_C_2_-AO-PA | 108.50 | 100 | U-spiked deionized water | [S21] |
| d | PAF-170-AO | 702.00 | 85 | U-spiked simulated seawater | [S22] |
| e | SA-DNA | 189.50 | 60 | U-spiked simulated seawater | [S23] |
| f | COF-R_5_ | 253.70 | 50 | U-spiked seawater | [S24] |
| g | CMCS/P(AO-co-AM-Cu) | 962.40 | 32 | U-spiked seawater | [S25] |
| h | PAO/Alg NF | 803.77 | 32 | U-spiked seawater | [S26] |
| i | AO-PIM-1 | 345.94 | 32 | U-spiked water | [S27] |
| j | DNA-UEH | 16.15 | 16 | U-spiked simulated seawater | [S28] |
| k | PPH-OP | 139.47 | 16 | U-spiked simulated seawater | [S29] |
| l | TPM | 933.32 | 450 | U-spiked deionized water | [S30] |
| m | g-C_3_N_4_/GO | 322.00 | 80 | U-spiked deionized water | [S31] |
| n | PAO@MXene | 639.98 | 60 | U-spiked deionized water | [S32] |
| o | CPP | 541.20 | 35 | U-spiked filtered natural seawater | [S33] |
| p | Anti-GO-BPG-GLACS | 599.00 | 32 | U-spiked water | [S34] |
| q | TTh-COF-AO | 682.10 | 20 | U-spiked seawater | [S35] |
| r | PGM-1 | 443.20 | 20 | U-spiked deionized water | [S36] |
| s | COF-4P | 449.30 | 20 | U-spiked seawater | [S37] |
| t | COF 2-Ru-AO | 382.00 | 19 | U-spiked seawater | [S38] |
| u | MOF/BPQD | 472.90 | 16 | U-spiked simulated seawater | [S39] |
| v | Zr/Ti-MOF-25 | 438.80 | 16 | U-spiked simulated seawater | [S40] |
| w | LB-COF | 320.00 | 8 | U-spiked water | [S41] |
| / | **CNF-A** | **920.12** | **128** | **U-spiked deionized water** | **This work** |

**Table S7** The original data of adsorption amount of competitive metal ions under visible light irradiation for 24 h

| **Metal ions** | **Initial concentration (ppb)** | **Adsorption amount (mg g^-1^)** | **SE** | **K_d_**  **(×10^4^ mL g^-1^)** |
| --- | --- | --- | --- | --- |
| U | 330 | 30.36 | 0.62 | 4.530 |
| V | 200 | 13.12 | 0.60 | 0.015 |
| Fe | 300 | 6.89 | 0.35 | 0.003 |
| Zn | 400 | 2.39 | 0.14 | 0.001 |
| Cu | 60 | 0.55 | 0.05 | 0.004 |
| Ni | 100 | 0.45 | 0.06 | 0.001 |
| Pb | 3 | 0.34 | 0.04 | 0.283 |
| Co | 5 | 0.18 | 0.04 | 0.005 |

Note: K_d_ is the selective coefficient calculated based on the equation K_d_=(C_adsorbent_)/(C_solution_).

**Table S8** The original data of U adsorption amount and elution efficiency in a 6 successive adsorption-desorption cycles under visible light irradiation

| **Cycle number** | **U adsorption capacity (mg g^-1^)**  **(Initial conc.=128 ppm)** | | | **Average (mg g^-1^)** | **SE** |
| --- | --- | --- | --- | --- | --- |
| 1 | 900.40 | 922.03 | 934.66 | 919.03 | 17.33 |
| 2 | 882.71 | 915.09 | 904.85 | 900.88 | 16.55 |
| 3 | 901.01 | 891.89 | 867.14 | 886.68 | 17.53 |
| 4 | 826.93 | 860.54 | 839.11 | 842.19 | 17.02 |
| 5 | 843.98 | 830.28 | 810.58 | 828.28 | 16.79 |
| 6 | 819.56 | 829.41 | 797.71 | 815.56 | 16.22 |
| **Cycle number** | **U elution efficiency (%)** | | | **Average (%)** | **SE** |
| 1 | 103.17 | 95.57 | 99.93 | 99.56 | 3.81 |
| 2 | 95.7 | 99.56 | 101.68 | 98.98 | 3.03 |
| 3 | 97.01 | 102.59 | 92.93 | 97.51 | 4.85 |
| 4 | 88.86 | 92.61 | 98.33 | 93.27 | 4.77 |
| 5 | 90.02 | 94.41 | 85.63 | 90.02 | 4.39 |
| 6 | 83.1 | 92.16 | 87.36 | 87.54 | 4.53 |

**Table S9** The comprehensive performances of COF-based aerogels used for UES

| **Aerogels** | **U Adsorption**  **capacity (mg g^-1^)** | **K_d_**  **(×10^4^ mL g^-1^）** | **COF content (%)** | **S_BET_ (m^2^ g^-1^)** | **U adsorption rate (%)** | **Refs.** |
| --- | --- | --- | --- | --- | --- | --- |
| **CNF-A** | **920.12** | **4.53** | **100.00** | **396.15** | **89.83** | **This work** |
| TpTHA/CNF | 177.90 | 1.90 | 34.00 | 28.15 | 74.13 | S42 |
| BCCOF-SO_3_NH_4_ | 883.44 | 7.00 | 42.60 | 70.77 | 86.42 | S43 |
| BHMS3 | 402.30 | 0.02 | 23.08 | 634.00 | 11.50 | S44 |
| PCA_10.0_ | 361.40 | 0.45 | 90.91 | 188.27 | 61.61 | S45 |

Note: K_d_ is the selective coefficient calculated based on the equation K_d_=(C_adsorbent_)/(C_solution_).

**Supplementary References**

1. J. Zou, A. O. Sbodio, B. Blanco-Ulate, L. Wang, G. Sun, Novel Robust, Reusable, Microbial-Resistant, and Compostable Protein-Based Cooling Media. Adv. Funct. Mater. **32**(26), 2201347 (2022). <https://doi.org/10.1002/adfm.202201347>
2. Z. Zhang, Y. Si, G. Sun, Photoactivities of Vitamin K Derivatives and Potential Applications as Daylight-Activated Antimicrobial Agents. ACS Sustain. Chem. Eng. **7**(22), 18493-18504 (2019). <https://doi.org/10.1021/acssuschemeng.9b04449>
3. Y. Si, Z. Zhang, W. Wu, Q. Fu, K. Huang et al., Daylight-driven rechargeable antibacterial and antiviral nanofibrous membranes for bioprotective applications, Sci. Adv. **4**(3), eaar5931 (2018). <https://doi.org/10.1126/sciadv.aar5931>
4. A. Halder, S. Kandambeth, B. P. Biswal, G. Kaur, N. C. Roy et al., Decoding the Morphological Diversity in Two Dimensional Crystalline Porous Polymers by Core Planarity Modulation. Angew. Chem. Int. Ed. **55**(27), 7806-7810 (2016). <https://doi.org/10.1002/anie.201600087>
5. A. F. M. El-Mahdy, Y. Hung, T. H. Mansoure, H. Yu, T. Chen et al., A Hollow Microtubular Triazine- and Benzobisoxazole-Based Covalent Organic Framework Presenting Sponge-Like Shells that Functions as a High-Performance Supercapacitor. Chem. Asian J. **14**(9), 1429-1435 (2019). <https://doi.org/10.1002/asia.201900296>
6. W. Kong, W. Jia, R. Wang, Y. Gong, C. Wang et al., Amorphous-to-crystalline transformation toward controllable synthesis of fibrous covalent organic frameworks enabling promotion of proton transport. Chem. Commun. **55**(1), 75-78 (2019). <https://doi.org/10.1039/c8cc08590k>
7. D. Rodríguez-San-Miguel, A. Abrishamkar, J. A. R. Navarro, R. Rodriguez-Trujillo, D. B. Amabilino et al., Crystalline fibres of a covalent organic framework through bottom-up microfluidic synthesis. Chem. Commun. **52**(59), 9212-9215 (2016). <https://doi.org/10.1039/c6cc04013f>
8. S. Wang, Z. Zhang, H. Zhang, A. G. Rajan, N. Xu et al., Reversible Polycondensation-Termination Growth of Covalent-Organic-Framework Spheres, Fibers, and Films. Matter **1**(6), 1592-1605 (2019). <https://doi.org/10.1016/j.matt.2019.08.019>
9. X. Ma, K. R. Meihaus, Y. Yang, Y. Zheng, F. Cui et al., Photocatalytic Extraction of Uranium from Seawater Using Covalent Organic Framework Nanowires. J. Am. Chem. Soc. **146**(33), 23566-23573 (2024). <https://doi.org/10.1021/jacs.4c07699>
10. T. Ma, E. A. Kapustin, S. X. Yin, L. Liang, Z. Zhou et al., Single-crystal x-ray diffraction structures of covalent organic frameworks. Science **361**(6397), 48-52 (2018). <https://doi.org/10.1126/science.aat7679>
11. W. E. Morton, J. W. S. Hearle, Fibre length. Phys. Prop. Text. Fibres **1**, 134-162 (2008). <https://doi.org/10.1533/9781845694425.134>
12. Y. Elmogahzy, R. Farag, 7-Tensile properties of cotton fibers: Importance, research, and limitations. Tens. Prop Cot. Fibers **1**, 223-273 (2018). <https://doi.org/10.1016/B978-0-08-101272-7.00007-9>
13. L. Lu, X. Liu, Y. Sun, S. Wang, J. Liu et al., Silk-Fabric Reinforced Silk for Artificial Bones. Adv. Mater. **36**(23), 230874 (2024). <https://doi.org/10.1002/adma.202308748>
14. B. A. Alshammari, M. Hossain, A. M. Alenad, A. G. Alharbi, B. M. Alotaibi, Experimental and Theoretical Analysis of Mechanical Properties of Graphite/Polyethylene Terephthalate Nanocomposites. Polymers **14**(9), 1718 (2022). <https://doi.org/10.3390/polym14091718>
15. I. C. Yeh, B. C. Rinderspacher, J. W. Andzelm, L. T. Cureton, J. La Scala, Computational study of thermal and mechanical properties of nylons and bio-based furan polyamides. Polymer **55**(1), 166-174 (2014). <https://doi.org/10.1016/j.polymer.2013.11.009>
16. D. Sawai, Y. Fujii, T. Kanamoto, Development of oriented morphology and tensile properties upon superdawing of solution-spun fibers of ultra-high molecular weight poly(acrylonitrile). Polymer **47**(12), 4445-4453 (2006). <https://doi.org/10.1016/j.polymer.2006.03.067>
17. Y. L. Yu, X. Q. Xu, C. D. Lu, T. H. Zhang, Y. Ma, Investigation on the microstructural and mechanical properties of a Polytetrafluoroethylene thin film by radio frequency magnetron sputtering. Thin Solid Films **712**, 138302 (2020). <https://doi.org/10.1016/j.tsf.2020.138302>
18. D. Gregor-Svetec, F. Sluga, High modulus polypropylene fibers. I. Mechanical properties. J. Appl. Polym. Sci. **98**(1), 1-8 (2005). <https://doi.org/10.1002/app.21990>
19. W. Zhang, M. Wu, Y. Xin, H. Liu, F. Li et al., Comparative analysis of seawater uranium extraction materials: Toward the development of bio-based and biomimetic materials. Coord. Chem. Rev. **534**, 216589 (2025). <https://doi.org/10.1016/j.ccr.2025.216589>
20. Y. Yuan, S. Feng, L. Feng, Q. Yu, T. Liu et al., A Bio-inspired Nano-pocket Spatial Structure for Targeting Uranyl Capture. Angew. Chem. Int. Ed. **59**(11), 4262-4268 (2020). <https://doi.org/10.1002/anie.201916450>
21. D. Zhang, L. Liu, B. Zhao, X. Wang, H. Pang et al., Highly efficient extraction of uranium from seawater by polyamide and amidoxime co-functionalized MXene. Environ. Pollut. **317**, 120826 (2023). <https://doi.org/10.1016/j.envpol.2022.120826>
22. Z. Li, Q. Meng, Y. Yang, X. Zou, Y. Yuan et al., Constructing amidoxime-modified porous adsorbents with open architecture for costeffective and efficient uranium extraction. Chem. Sci. **11**(18), 4747-4752 (2020). <https://doi.org/10.1039/d0sc00249f>
23. W. Zhang, Y. Xin, Y. Fa, F. Li, Y. Liu et al., SA-DNA hydrogel microspheres for Ultra-Selective uranyl (VI) extraction from seawater. Chem. Eng. J. **495**, 153690 (2024). <https://doi.org/10.1016/j.cej.2024.153690>
24. Y. Xie, Y. Wu, X. Liu, M. Hao, Z. Chen et al., Rational design of cooperative chelating sites on covalent organic frameworks for highly selective uranium extraction from seawater. Cell Reports Phys. Sci. **4**(1), 101220 (2023). <https://doi.org/10.1016/j.xcrp.2022.101220>
25. Y. Zhang, Y. Wang, Z. Dong, Y. Wang, Y. Liu et al., Boosting uranium extraction from Seawater by micro-redox reactors anchored in a seaweed-like adsorbent. Nat. Commun. **15**, 9124 (2024). <https://doi.org/10.1038/s41467-024-53366-3>
26. X. Xu, Y. Yue, D. Cai, J. Song, C. Han et al., Aqueous Solution Blow Spinning of Seawater-Stable Polyamidoxime Nanofibers from Water-Soluble Precursor for Uranium Extraction from Seawater. Small Methods **4**(12), 2000558 (2020). <https://doi.org/10.1002/smtd.202000558>
27. L. Yang, H. Xiao, Y. Qian, X. Zhao, X. Kong et al., Bioinspired hierarchical porous membrane for efficient uranium extraction from seawater. Nat. Sustain. **5**, 71-80 (2022). <https://doi.org/10.1038/s41893-021-00792-6>
28. Y. Yuan, T. Liu, J. Xiao, Q. Yu, L. Feng et al., DNA nano-pocket for ultra-selective uranyl extraction from seawater. Nat. Commun. **11**, 5708 (2020). <https://doi.org/10.1038/s41467-020-19419-z>
29. Y. Yuan, Q. Yu, M. Cao, L. Feng, S. Feng et al., Selective extraction of uranium from seawater with biofouling-resistant polymeric peptide. Nat. Sustain. **4**, 708 (2021). <https://doi.org/10.1038/s41893-021-00709-3>
30. B. Zhang, X. Shan, J. Yu, H. Zhang, K. Tawfik Alali et al., Facile synthesis of TiO_2_-PAN photocatalytic membrane with excellent photocatalytic performance for uranium extraction from seawater. Sep. Purif. Technol. **328**, 125026 (2024). <https://doi.org/10.1016/j.seppur.2023.125026>
31. Y. Liao, B. Yuan, D. Zhang, J. Zhang, X. Wang et al., Fabrication of Heterostructured Metal Oxide/TiO_2_ Nanotube Arrays Prepared via Thermal Decomposition and Crystallization. Inorg. Chem. **57**(16), 10249-10256 (2018). <https://doi.org/10.1021/acs.inorgchem.8b01483>
32. M. Fu, C. Huang, L. Ma, Y. Yao, J. Chen et al., Solar enhanced uranium extraction from seawater with the efficient strategy of MXene loaded nano-porous polyamidoxime membrane. Sep. Purif. Technol. **332**, 125803 (2024). <https://doi.org/10.1016/j.seppur.2023.125803>
33. W. R. Cui, C. R. Zhang, R. P. Liang, J. D. Qiu, Covalent organic framework hydrogels for synergistic seawater desalination and uranium Extraction. J. Mater. Chem. A **9**(45), 25611-25620 (2021). <https://doi.org/10.1039/d1ta06732j>
34. T. Li, X. Lin, Z. Zhang, L. Yang, Y. Qian et al., Photothermal-Enhanced Uranium Extraction from Seawater: A Biomass Solar Thermal Collector with 3D Ion-Transport Networks. Adv. Funct. Mater. **33**(19), 2212819 (2023). <https://doi.org/10.1002/adfm.202212819>
35. F. Yu, C. Li, W. Li, Z. Yu, Z. Xu et al., Π-Skeleton Tailoring of Olefin-Linked Covalent Organic Frameworks Achieving Low Exciton Binding Energy for Photo-Enhanced Uranium Extraction from Seawater. Adv. Funct. Mater. **34**(1), 2307230 (2024). <https://doi.org/10.1002/adfm.202307230>
36. Z. Liu, K. Feng, X. Zhang, L. Fu, J. Ren et al., Enhanced uranium extraction using a nanostructured photothermal hydrogel membrane. Chem. Eng. J. **498**, 155423 (2024). <https://doi.org/10.1016/j.cej.2024.155423>
37. H. Yang, M. Hao, Y. Xie, X. Liu, Y. Liu et al., Tuning Local Charge Distribution in Multicomponent Covalent Organic Frameworks for Dramatically Enhanced Photocatalytic Uranium Extraction. Angew. Chem. Int. Ed. **62**(30), e202303129 (2023). <https://doi.org/10.1002/anie.202303129>
38. M. Hao, Y. Xie, X. Liu, Z. Chen, H. Yang et al., Modulating Uranium Extraction Performance of Multivariate Covalent Organic Frameworks through Donor-Acceptor Linkers and Amidoxime Nanotraps. JACS Au **3**(1), 239-251 (2023). <https://doi.org/10.1021/jacsau.2c00614>
39. M. Chen, T. Liu, X. Zhang, R. Zhang, S. Tang et al., Photoinduced Enhancement of Uranium Extraction from Seawater by MOF/Black Phosphorus Quantum Dots Heterojunction Anchored on Cellulose Nanofiber Aerogel. Adv. Funct. Mater. **31**(22), 2100106 (2021). <https://doi.org/10.1002/adfm.202100106>
40. T. Liu, S. Tang, T. Wei, M. Chen, Z. Xie et al., Defect-engineered metal-organic framework with enhanced photoreduction activity toward uranium extraction from seawater. Cell Reports Phys. Sci. **3**(5), 100892 (2022). <https://doi.org/10.1016/j.xcrp.2022.100892>
41. Y. Zhao, S. Li, G. Fu, H. Yang, S. Li et al., Construction of Layer-Blocked Covalent Organic Framework Heterogenous Films via Surface-Initiated Polycondensations with Strongly Enhanced Photocatalytic Properties. ACS Cent. Sci. **10**(4), 775-781 (2024). <https://doi.org/10.1021/acscentsci.3c01195>
42. M. Li, B. Qing, H. Luo, W. Gao, Q. Shou et al., Recyclable covalent organic frameworks/cellulose aerogels for efficient uranium adsorption. Int. J. Biol. Macromol. **282**, 137156 (2024). <https://doi.org/10.1016/j.ijbiomac.2024.137156>
43. M. Li, L. Sun, W. Gao, B. Qing, H. Yao et al., In-situ bioprocessing of bacterial cellulose aerogel with covalent organic frameworks for enhanced uranium extraction. Sep. Purif. Technol. **355**, 129654 (2025). <https://doi.org/10.1016/j.seppur.2024.129654>
44. W. Cui, C. Zhang, R. Liang, J. Liu, J. Qiu, Covalent Organic Framework Sponges for Efficient Solar Desalination and Selective Uranium Recovery. ACS Appl. Mater. Interfaces **13**(27), 31561-31568 (2021). <https://doi.org/10.1021/acsami.1c04419>
45. B. Fan, Y. Si, J. Yu, X. Wang, P. Tang, Multi-Interfacial Interaction Engineered Underwater Superelastic Covalent Organic Framework Aerogels for Photoinduced Uranium Extraction. Chem. Eng. J. **498**, 155756 (2024). <https://doi.org/10.1016/j.cej.2024.155756>
